# Structure, dynamics and kinetics of two-component Lantibiotic Lichenicidin

**DOI:** 10.1371/journal.pone.0179962

**Published:** 2017-06-27

**Authors:** Alejandra de Miguel, Rafael Tapia-Rojo, Tillmann Utesch, Maria Andrea Mroginski

**Affiliations:** 1 Department of Biophysical Chemistry, Institut für Chemie, Technische Universität Berlin, Berlin, Germany; 2 Departamento de Física de la Materia Condensada, Universidad de Zaragoza, Zaragoza, Spain; 3 Instituto de Biocomputación y Física de Sistemas Complejos, Universidad de Zaragoza, Zaragoza, Spain; Hong Kong University of Science and Technology, HONG KONG

## Abstract

Two variants of the two-component Lantibiotic Lichenicidin, produced by the strains *B*. *Licheniformis* VK21 and I89 (Lchα/ Lchβ and Bliα/ Bliβ peptides, respectively) have been investigated by means of 2 μs-long all-atom molecular dynamics simulations combined with Markov State Models. This rigorous statistical analysis enabled to evaluate the dynamic and kinetic properties of the aforementioned systems which are not accessible via experimental techniques. The structural flexibility characteristic of these small peptides is understood by a delicate equilibrium between random coil, α-helices and β-sheet structures. The undergoing secondary structure transitions from an α-helix to a β-sheet observed for Lchα and Lchβ peptides, were not present in the Bliα component and provide new insights to understand their mechanism of action.

## Introduction

Antimicrobial peptides (AMPs) are synthesized by a wide diversity of prokaryotic and eukaryotic microorganisms (e.g. bacteria, plants, amphibians or mammals [1–7]) being their first line of defense in the innate immune system. AMPs produced by bacteria are commonly called bacteriocins [8].

Bacteriocins exclusively kill other bacteria. Bacteriocins produced by Gram- negative bacteria are classified in two families, the colicins and the microcins. Colicins show a higher molecular mass (30–80 kD) in comparison to microcins (1–10 kD). Their segregation is promoted by different factors: from a response due to DNA damages in the case of the colicins or stress conditions characterized by low levels of nutrients for microcins [9]. Bacteriocins produced by Gram- positive bacteria are small peptides (2–5 kD) whose distinctive size can be most likely attributed to their capability of permeabilizing the peptidoglycan layer. Lantibiotics is a term used to denote bacteriocins produced by Gram- positive bacteria that contain lanthionine and/or methyllanthionine residues [10].

In general, AMPs are positively charged and characterized by a high degree of amphipathicity [11]. Although they can adopt all types of secondary structures, α-helix and β-sheet are the most common ones [12]. In solution, many AMPs manifest unordered structures [13], which then fold into amphipathic α-helix or β-sheet conformations upon interaction with membranes or with other reaction partners [13]. The two-component Lichenicidin lantibiotic may belong to this last class.

This work is focused on the study of Lichenicidin lantibiotic produced by the bacteria *Bacillus Licheniformis*. Lantibiotics are ribosomally synthesized as propeptides which are then posttranslationally modified (RiPPs) to their biologically active forms [14]. During the posttranslational modifications unique structural amino acids are introduced, such as the thioether amino acids lanthionine (Lan) and methyllanthionine (MLan). Furthermore, dehydrated amino acids, such as dehydroalanine (Dha) and dehydrobutyrine (Dhb), are common constituents of the peptide backbone.

So far, it has been shown that several strains of *Bacillus Licheniformis* produce Lichenicidin. Among them, the isogenic strains ATCC 14580 and DSM 13 [15] as well as the VK21 [16] strain have been thoroughly characterized in the NCBI database [17]. However, only for the mature lantibiotic peptides generated by *B*. *Licheniformis* VK21, named as Lchα and Lchβ, three dimensional structures are available. The corresponding structures were solved by NMR spectroscopy in methanol solution (pdb-entries: 2KTN and 2KTO) [16]. According to these structures, Lchα consists of a flexible loop connecting the N- and C- terminal domains which is stabilized by a thioether bridge between residues 11 and 21. Conversely, Lchβ folds into an α-helix with highly mobile N- and C- terminal domains (S1 Fig). However, the considerable signal doubling observed in the NMR spectra is a strong indication for conformational exchange in the millisecond time range. This is particularly true for the Lchα, for which a root-mean-square deviation of the atomic positions of 6.45 ± 1.79 Å has been estimated. [16] Furthermore, since the NMR experiments were performed in methanol which is known to enhance secondary structure formation [18], the structural properties of these peptides in aqueous solution remain unsolved.

**Fig 1:**
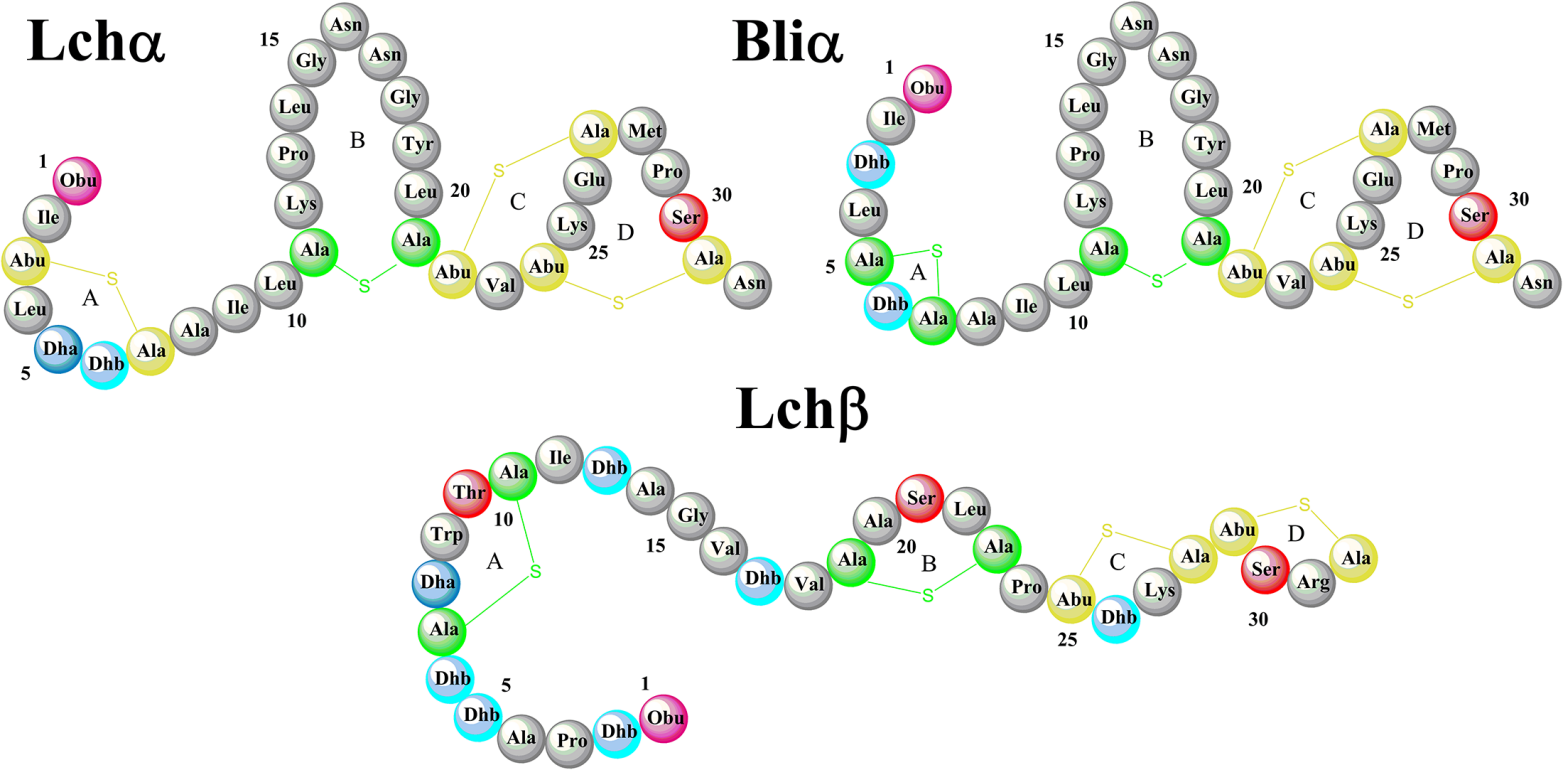
Lichenicidin Lantibiotic. Lichenicidin Lantibiotic is composed by Lchα and Lchβ peptides [16]. Bliα is the α-component produced by *B*. *Licheniformis* I89. Both α-peptides differ in the A-ring being β-peptides identical.

In addition to the *B*. *Licheniformis* strains mentioned above, it was later discovered that the strain I89 is also capable of producing Lichenicidin. [19] The mature lantibiotic peptides were named Bliα and Bliβ. However, on the basis of MS/MS spectroscopy [20], the amino acid sequence for the α-peptide was proposed to be different than that detected in the earlier cases. The main difference between the primary structures of Lchα and Bliα lies exclusively in the definition of the A-ring where the methyllanthionine bridge between residues 3 to 7 of Lchα is replaced by a short lanthionine bridge involving residues 5 and 7 in Bliα (Fig 1). However, no differences in the amino sequence between Bliβ and Lchβ were found. So far, the three dimensional structure of the Bliα peptide has not been solved.

In this study we employed atomistic molecular dynamics (MD) simulations to investigate the structural and dynamic properties of three lichenicidin peptides (Lchα, Lchβ and Bliα) in aqueous solution. This technique has been successfully employed to study a wide spectrum of biomolecules. [21–24] Given the complexity of the conformational space sampled for these flexible peptides, a rigorous statistical approach based on Markov States Models (MSMs) was carried out in order to accurately resolve the dynamics, generate free energy landscapes and predict the kinetics of these three peptides in aqueous solution.

## Materials and methods

### Definition of the potential energy function

CHARMM (Chemistry at Harvard Macromolecular Mechanics) is a well-established additive force field where the potential energy function (U(r)) is defined as a sum of pair interactions in the form of bonded and nonbonded terms described in Eq 1. [25–27]

$$\begin{array}{l} U\left(\bar{r}\right)=\sum_{bond}K_{b}{\left(b-b_{0}\right)}^{2}\;+\sum_{UB}K_{UB}{\left(S-S_{0}\right)}^{2}\;+\sum_{angle}K_{\theta }{\left(\theta -\theta_{0}\right)}^{2}\\ +\;\sum_{dihedral}K_{\chi }\left(1+\text{cos}\left(n\chi -\delta \right)\right)\;+\sum_{improper}K_{\varphi }\left(\varphi +\varphi_{0}\right){\;}^{2}\\ +\;\sum_{nonbond}\left[\varepsilon_{ij}\left[{\left(\frac{R_{ij}}{r_{ij}}\right)}^{12}-2{\left(\frac{R_{ij}}{r_{ij}}\right)}^{6}\right]+\frac{q_{i}q_{j}}{4\pi \varepsilon r_{ij}}\right] \end{array}$$ (1)

The bonded terms account for bond stretches (*b*), changes of the Urey- Brandley 1-3-distances (*S*), bond angles bending (θ), dihedral angle torsions (χ) and variations of improper angles (*φ*). These terms with the exception of the dihedral angle term are described by harmonic potentials associated with force constants K and equilibrium values *b*_*0*_, *S*_*0*_, *θ*_*0*_ and *φ*_*0*_, respectively. The dihedral angle term is described by a sinusoidal function with multiplicity *n* and phase shift *δ*. The Urey-Bradley (UB) component, a cross term involving angle bending using nonbonded 1,3 interactions, is optional. The UB parameters are seldomly defined for new atom types, since it has been demonstrated that its contribution to the total potential energy is not significant. The nonbonded terms include the Coulomb and van der Waals interactions. The electrostatic interactions are a function of the atomic partial charges *q*_*i*_ and *q*_j_ and their relative distance *r*_*ij*_ following Coulomb’s law. The van der Waals interactions are describes by means of a Lennard-Jones 6–12 potential with parameters *ε*_ij_ and *R*_*ij*_ for the atom pair *i* and *j* separated a distance *r*_*ij*_.

#### Force field parameters for dehydrobutyrine and dehydroalanine

**Fig 2:**
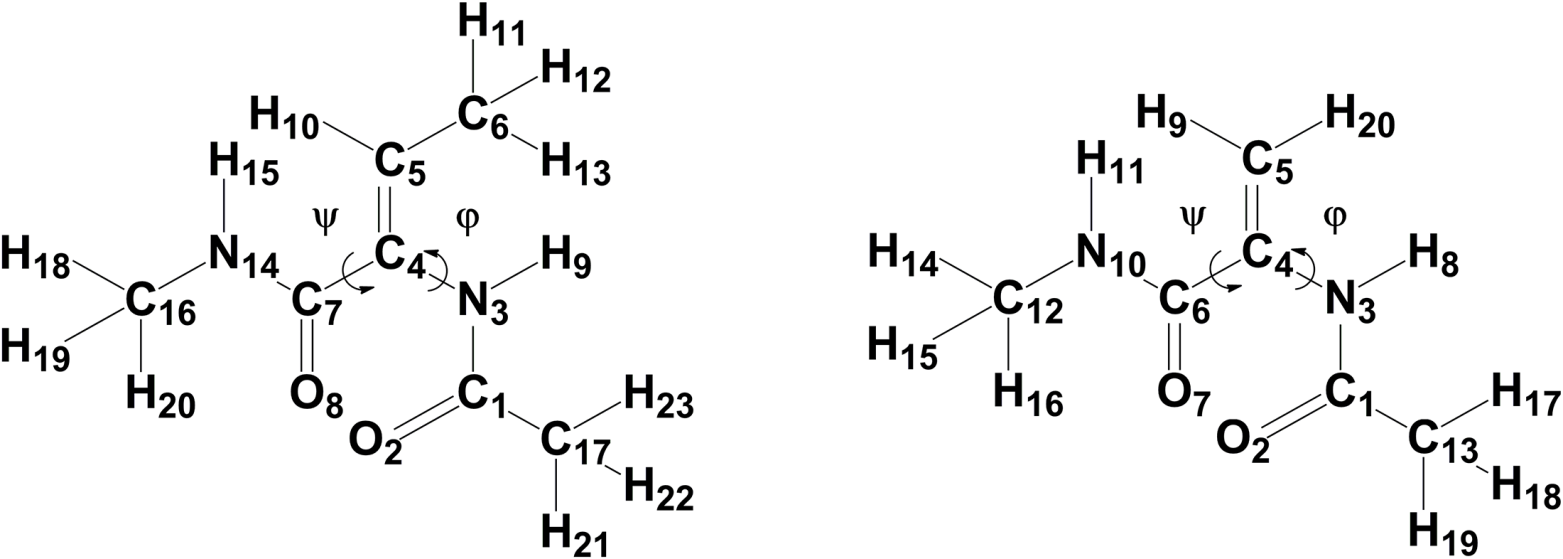
Target molecules. Structural formula for the dehydrobutyrine (left) and dehydroalanine (right) molecules.

In order to investigate the structural and dynamical properties of the Lichenicidin lantibiotic by means of classical MD simulations, CHARMM compatible force field parameters for dehydroamino acids are required. The first attempt to generate accurate CHARMM parameters for these amino acids was achieved by Thormann and Hofmann [28]. In their work, they applied the force field parameters for standard L-amino acids with a slight correction of the torsional potential term for the rotation around φ (torsional force constant was increased from 0.25 to 0.48) (Fig 2).

They showed that this modification was enough to reproduce the structural ab-initio and experimental data of the dehydroalanine peptide. Very recently, new CHARMM force field parameters for Dhb and Dha were reported by Turpin et al. [29]. In their work, the force field parametrization was performed using a well-established procedure based on the Paramchem interface (www.paramchem.org). Furthermore, parameters involving the Cα = Cβ double bond were reoptimized with the help of quantum mechanical calculations at the HF/6-31G* and MP2/6-31G* levels. However, a closer look at their parameter set for Dhb revealed that: a) only the E-isomer of Dhb was considered as target molecule, although it is the Z-isomer which is formed after posttranslational modifications of threonine [14] and, b) relevant force field parameters such as those involving the atom types CG2DC1 and CTD1, among others, are missing or are inconsistent. Given the uncertainty of this published data, new parameters were derived.

In our work, we followed the same procedure as reported by Turpin et al. [29], namely the use of the Paramchem.org server for generating CHARMM-compatible force field parameters for Dha and Dhb by analogy to other molecules. Both peptides were built with an acetylated N-terminus and N-methylamide at the C-terminus (Fig 2). In contrast to Turpin's parametrization procedure the Z-isomer of Dhb was taken into account as detected so far for all AMPs due to the posttranslational modifications [13,16]. Furthermore, following the improvement suggested by Thormann and Hofmann [28], the force constant for the torsional potential around atom types X-NP-CUA1-X (corresponding to the C7-C4-N3-C1dihedral) was set to 0.48. Further information on the force field parameters for Dha and Dhb is given in S1 File.

In order to compare the existing force field parameters for Dha and Dhb, the potential energy curves for partially constrained model compounds were computed at a quantum mechanical level and compared with those obtained at a molecular mechanical level using different sets of force field parameters (S2 and S3 Figs). For the quantum mechanical calculations, the geometry of the target molecules were initially optimized at the MP3/ HF (3-31G) / HF (6-31G*) level. Afterwards, dihedral scans around the ψ dihedral N14-C7-C4-N3 (N14-C6-C4-N3) and φ dihedral C7-C4-N3-C1 (C6-C4-N3-C1) of Dhb (Dha) were carried out in steps of 10° (S2 and S3 Figs, blue traces). The same molecular geometries were employed for the computation at the molecular mechanics level. The quantum chemical calculations were performed using the Gaussian 09 program [30] while the force field calculations were done with the NAMD Energy plugin in the VMD package [31]. The potential energy curves for the ψ dihedral angles of Dha and Dhb are practically the same for both peptides, with clearly defined global minima at about 30° and less pronounced local minima at ca. 140° and 220°, in agreement with the ab initio calculations. Conversely, the φ dihedral angle exhibits relatively flat minima with two energy barriers at about -20° and 170° for Dhb but only one barrier at about -10° for Dha. Independent of the parameter set used for the force field calculations, the height of the potential energy curves are overestimated compared to the corresponding MP2/6-31G* values. Since molecular dynamics simulation sample low energy states, emphasis was made in reproducing the energy wells.

### Model building

The starting geometries (Fig 1) for Lchα and Lchβ were extracted from the Protein Data Bank with the PDB-entries 2KTN and 2KTO, respectively [16]. Since these structures are derived from NMR measurements, the atomic coordinates of the representative conformer was chosen in both cases. The structure for Bliα was obtained using the atomic coordinates of Lchα as template. The aminobutyrate (Abu3) and the dehydroalanine (Dha5) were converted into dehydrobutyrine (Dhb3) and alanine (Ala5) residues, respectively. The latter was connected to Ala7 via a thioether bridge and further transformed into a lanthionine residue (residues 5 and 7). These *in silico* transformations were performed maintaining the correct stereochemistry using the software Gaussview [32].

Lchβ consists of a short peptide chain of 31 amino acids with two lanthionine bridges placed between the residues 7–11 and 19–23 closing rings A and B, respectively. Further, there are two methyllanthionine bridges between the residues 25–28 and 29–32, giving rise to the so called rings C and D, respectively. The structure consists of an α-helix between positions 9 to 18 and a N- terminal 2-oxobutyryl group. Furthermore, Lchβ is slightly positively charged, (net charge of +1).

Lchα is also a short peptide chain with only 31 amino acids, characterized by the presence of a single lanthionine bridge between residues 11–21 (closing ring B) and three methyllanthionine bridges between the residues: 3–7 (ring A), 22–27 (ring C) and 24–31 (ring D). In Bliα, however, the lanthionine bridge on ring A is built between the residue 5 and 7. Beyond residue Dhb6, both α-peptides exhibit the same sequence of amino acids. Furthermore, both peptides harbour a 2-oxobutyryl group at the N-termini. Although most lanthibiotics and antimicrobial peptides are positively charged, Lchα and Bliα exhibit a net charge of zero. The same electric property has been previously reported for α-component of lacticin or mersacidin. [33]

### Molecular dynamics (MD) simulations

In order to investigate the structural flexibility of two component lantibiotics, classical all-atom MD simulations of the α- and β-components of Lichenicidin produced by *B*. *Licheniformis* VK21 (Lchα, Lchβ) and the α-component produced by *B*. *Licheniformis* I89 (Bliα, Bliβ) were carried out. The Lichenicidin components produced by these two bacteria differ only in the structure of the ring A region of the α-peptide, while the β-peptides are identical (Fig 1). Thus, the MD simulations are restricted to Lchα, Lchβ and Bliα.

The three polypeptides were individually solvated in cuboid boxes of water molecules and ionized, according to the experimental studies of Mendo et al. [19], at pH 7.0 and with an ionic strength of 160 mM of NaCl. For this purpose, the SOLVATE and AUTOIONIZE plugins of VMD [31] were employed. The simulation systems for Bliα and Lchα solvated in water boxes (52 Å x 52 Å x 50 Å) contained about 4800 water molecules, 7 Na^+^ and 7 Cl^-^ ions. Due to its elongated shape, a larger water box (52 Å x 56 Å x 55 Å) was built for Lchβ harboring 6622 water molecules, 9 Na^+^ and 10 Cl^-^ ions.

The MD simulations were run with NAMD 2.9 program [34] under periodic boundary conditions and using the multistep integrator impulse-based Verlet-I (r-RESPA) [35]. For van der Waals (vdW) interactions and real space electrostatics a cut-off of 12 Å was applied, while long-range electrostatics was calculated with the Particle Mesh Ewald Summation [36]. The canonical amino acids were described with the CHARMM27 [25–27] force field while the dehydroamino acids Dha and Dhb were modeled using the CHARMM-compatible force field described in this work. All water molecules were treated with the TIP3P model [37]. To satisfy a time step of 2 fs, the SHAKE algorithm [38] was used to constrain all bond lengths between heavy and hydrogen atoms. At first, the energies of the three systems were minimized for 20000 steps with the conjugated gradient integrator stepwise decreasing harmonic constrains on all heavy atoms (from 25 to 5 kcal/(mol Å^2^)). Subsequently, the systems were heated during 25 ps using Langevin dynamics with a time step of 0.5 fs and decreasing position restraints on the heavy atoms from 5 to 2.5 kcal/(mol Å^2^). After heating, the solvated peptides were equilibrated for 60 ps. The harmonic constrains on the peptides were gradually released during the equilibration run until all atoms were allowed to move freely. Finally the dynamics for the three models were simulated with a time step of 2 fs for 2 μs at T = 300 K in an NPT ensemble under constant atmospheric pressure and temperature using the Langevin Piston method with a damping constant of 0.01 fs^-1^ [39]. In all production runs, the energies were evaluated at intervals of 0.5 ps while coordinate trajectories at intervals of 2 ps.

In order to simplify the analysis of the MD trajectories, secondary structure evolution plots were generated with the STRIDE program [40] implemented in the VMD package [31]. This code is able to identify secondary structure elements in a protein structure by means of a knowledge- based algorithm using information on backbone torsional angles and hydrogen bond energy. [40]

### Markov State Models (MSM)

The equilibrium ensemble of each peptide was described by building the corresponding Markov State Model (MSM) from the MD equilibrium trajectories [41–42]. The MSM was constructed with a two-step protocol, first calculating the microstate network (Conformational Markov network [43]) -performing a geometric discretization of the state space of the system-, second by lumping these microstates into kinetically significant clusters, from which physical insight could be obtained. Time-lagged Independent Analysis (TICA) [44] was performed on the C_α_ of the MD trajectories, defining the state space as the three first Independent Components (TICs), providing a kinetically meaningful dimension. Each TIC was discretized into 30 bins of equal volume. The microstate network was built from the MD trajectories, counting the occupation of each state *π*_*i*_ and calculating the transition matrix *T*_*ij*_, which measures the probability of going from state *i* to state *j* within time *τ* (here *τ* = 1 ns). For the three analyzed peptides, the obtained transition matrix is ergodic and fulfills detailed balance (microscopic reversibility):

$$\pi_{i}T_{ij}=\pi_{j}T_{ji}$$ (2)

Since the microstate network is typically made of a large number of nodes (~10^4^), extraction of any meaningful information from it is tedious. In this regard, it is convenient to lump microstates with some criteria in order to provide a coarse-grained description of the free energy landscape of the system. To do that, the Stochastic Steepest Descent algorithm [43] was applied, calculating the basins of attraction of the system, *i*.*e*. groups of nodes whose probability flux converges into a single node (minimum). The basins of attraction can be referred to as the macrostates of the system, and they are in this way defined according to the kinetic properties of the system, which allows for an association with the free energy minima in the free energy landscape of the system. This coarse-grained representation has a new associated transition matrix *T*_*ij*_ -which can be calculated from the microstate in a straightforward way- together with the population of each basin *π*_*i*_.

Chapman-Kolmogorov test was used to test the quality of the MSMs of the threepeptides. [41]. S5 Fig in supplementary materials shows the implied timescales (or relaxation timescales), plotted for lag times of 1, 2, 3, 5 and 10 ns. Clearly, all timescales level, proving the validity of the used MSMs with lag time of 1 ns.

Additionally, free energy differences between states *i* and *j* were calculated as

$$\Delta {\text{G}}_{\text{ij}}=-k_{B}\text{Tlog}\frac{\pi_{i}}{\pi_{j}}$$ (3)

while the mean escape time from each basin *i* was computed as

$$t_{e}=\frac{\tau }{\left(1-{\text{T}}_{ii}\right)}.$$ (4)

The entropy of each macrostate (*i*) was compute according to Shannon’s information theory assuming a uniform distribution of mircrostates [45] as

$$S_{\alpha }=-\overset{}{\underset{i\in \alpha }{\sum }}p_{i}logp_{i}$$ (5)

where *p*_*i*_ is the population of microstate *i* and the sum runs over all microstates contained in macrostate α.

Additionally, molecular properties from each state, such as, root mean square deviation (RMSD) and root mean square fluctuations (RMSF) were estimated from the marginal distributions of the corresponding macrostate.

## Results and discussion

**Fig 3:**
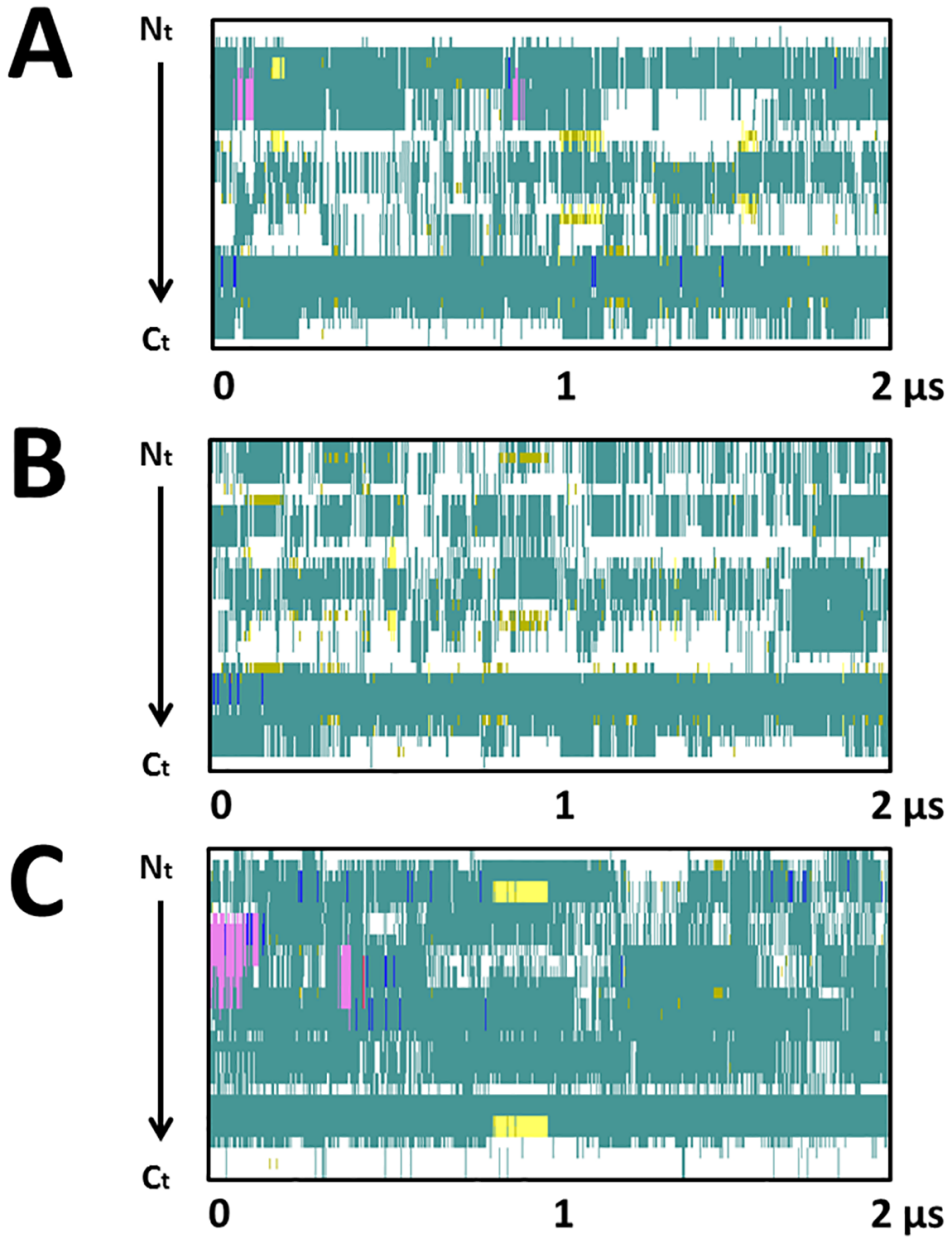
Secondary structure evolution of the peptides. Evolution of the secondary structure during the 2 *μs* MD trajectory is shown for (A) Lchα, (B) Bliα and (C) Lchβ. The following color code is used: pink for α-helix; yellow for β-sheet, blue for a 3_10_ helix, red for a π-helix, turquoise for a turn, light green for an isolated bridge and white for random coil. These plots were generated with the STRIDE tool of the VMD software [31, 40]

**Fig 4:**
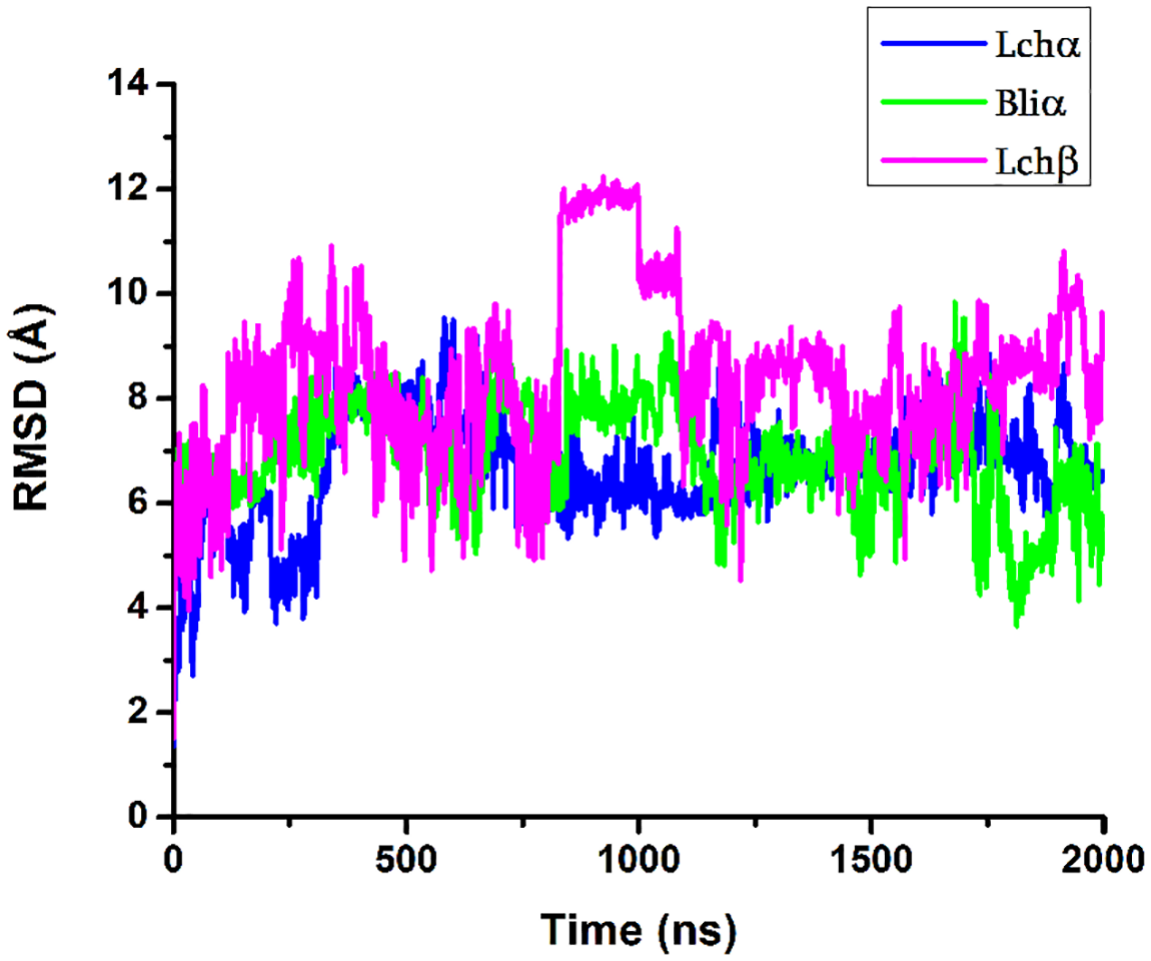
Root mean square deviation of lichenicidin. The RMSD with respect to initial configuration of the backbone atoms for Lchα (blue color); Bliα (green color); Lchβ (magenta) peptides is plotted. Terminal residues were excluded for the evaluation.

The structural and dynamical properties of the two-component Lichenicidin lantibiotic in aqueous solution were studied by means of classical all atoms MD simulation. The evolution of the secondary structures of these three peptides during the 2 *μs*-long MD simulation is depicted in Fig 3 while the change of the RMSD of the backbone heavy atoms with respect to the corresponding initial structures is shown in Fig 4.

The dynamical behaviors of the three peptides reflected in the evolution of the secondary structure predicted by the MD simulations, differ from each other. In general, we observe that the three peptides show basically flexible turns and random coil conformations (white and turquoise regions in Fig 3) and tend to form sporadically β-sheet structures (yellow lines in Fig 3). Interestingly, only Lchα and Lchβ have the ability to adopt, in addition, α-helical structures in the nanosecond time scale, as indicated by the pink regions in Fig 3. This property is not predicted for Bliα for which a single short lasting 3_10_-helix resulting from sporadic hydrogen bonded interaction between Abu24 and Glu27 is formed. The higher rigidity at the N-terminal region of Lchα compared to Bliα is most likely a consequence of the methyllanthionine bridge connecting residues 3 and 7 present in the former and absent in the latter peptide. Indeed, the RMSF of the α-carbons averaged over the entire peptide of Bliα is 5.16 ±1.51 Å, are slightly higher compared to the RMSF predicted for Lchα of only 4.77±1.32 Å. In the case of Lchβ, the lanthionine bridge between residues 7 and 11, serve as stabilizing structural element.

Analysis of the RMSD plots depicted in Fig 4 reflected large conformational changes of the peptides-backbone compared to the initial conformations with RMSD values ranging from 4 to 10 Å for the two α-components and between 2 to 13 Å for the β-component. For Lchα, the computed average RMSD value of about 6.7 ± 1.0 Å is comparable to the experimental value of 6.45 ± 1.79 Å estimated via NMR spectroscopy. [16] For the Lchβ peptide, however, larger differences were encountered. The average RMSD value of about 9.0 ± 1.7 Å predicted for the β-component is significantly larger than the experimental value of only 3.1 ± 1.0 Å [16]. This large difference between calculated and experimental RMSD values of the Lchβ peptide can be most likely attributed to solvent effects since, the NMR experiments were performed in methanol solution which is know to stabilize secondary structure elements [18].

Due to the complexity of the RMSD traces and the high flexibility of the three peptides in aqueous solution, it was not possible to clearly identify and characterize relevant kinetic states. Thus, in order to capture the essential features characterizing the kinetics properties and dynamical behavior of these antibiotics, Markov State Models (MSM) were generated. The development of these models as well as their analysis and interpretation is discussed in the following.

### Construction of Markov State Models

The MSMs of Lchα, Bliα, Lchβ peptides were developed according to the description detailed in materials and methods. For Lchα, a microstate network made of 3144 nodes connected through 8452 links was obtained. After applying the SSD algorithm, the network was clustered into a total of 35 macrostates, connected through 345 links. The microstate network was refined by eliminating nodes with an occupation *π*_*i*_<10^-4^ in order to avoid pathological or extremely rare states. The definitive description of the Lchα peptide is a network composed by 14 configurations connected through 54 allowed transitions including auto-links.

The MSMs of Bliα were built following the same protocol as used for Lchα. In this case, a microstate network of 1424 nodes connected through of 3560 links was generated, which were further reduced to 38 nodes connected through 95 transitions, via the SDD algorithm. After refinements indicated above, we obtain the final description of the Bliα peptide as composed by 9 configurations with 48 allowed transitions.

Finally, the MSM for Lchβ was also calculated. In this case, the microstate network of 725 nodes connected through of 1805 links was obtained. Following the same SDD procedure as before, the unrefined network of macrostates was made up of 25 nodes connected through 625 transitions ending up in a simple refined network with 7 configurations and 25 transitions.

**Fig 5:**
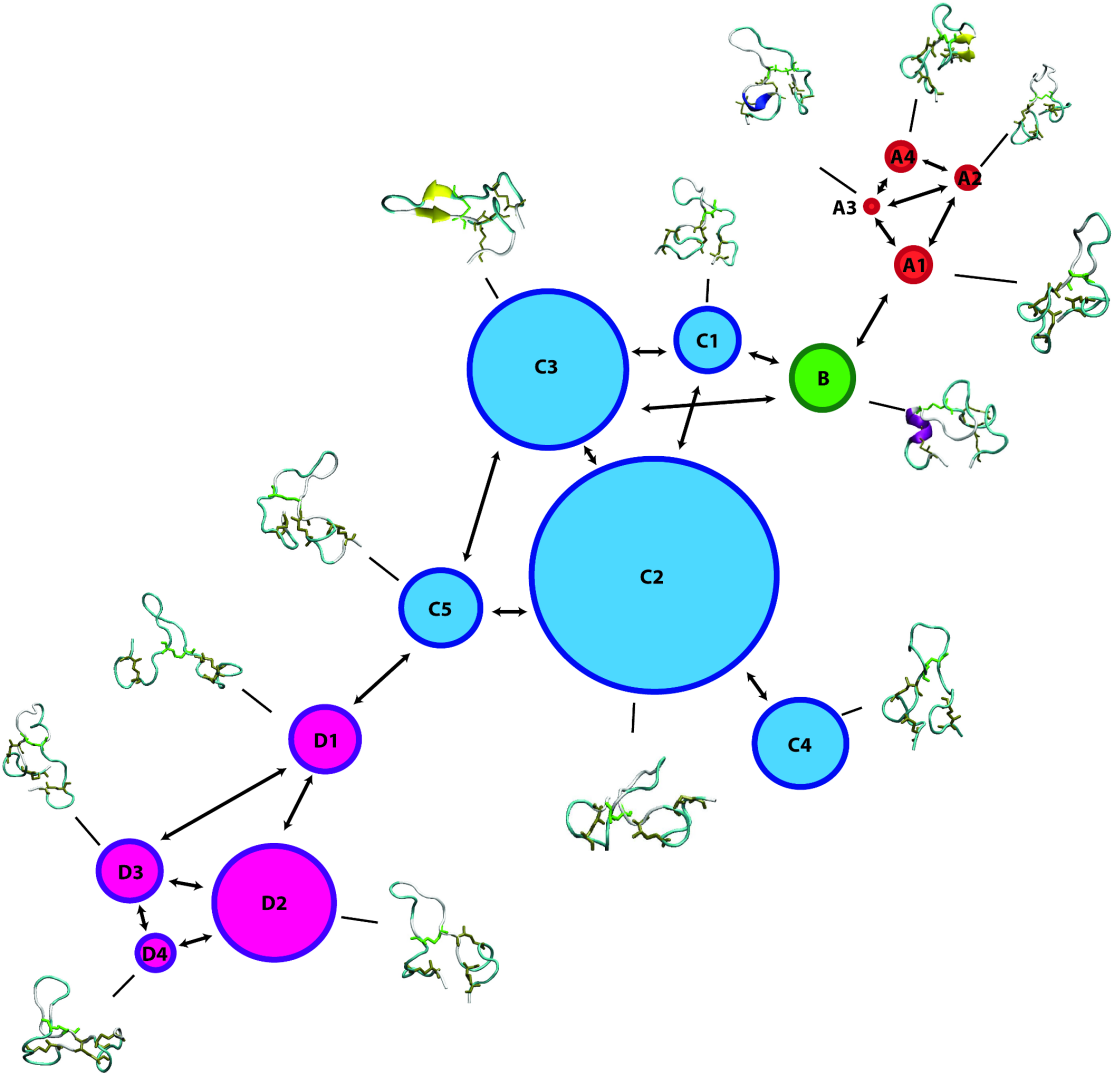
Markov State Model of Lchα peptide. MSM network is based on the information listed in Table 1. The 14 states are depicted as beads with size proportional to their occupation, arrows represent allowed transitions between states according to the corresponding transition matrix in Fig 8.

**Fig 6:**
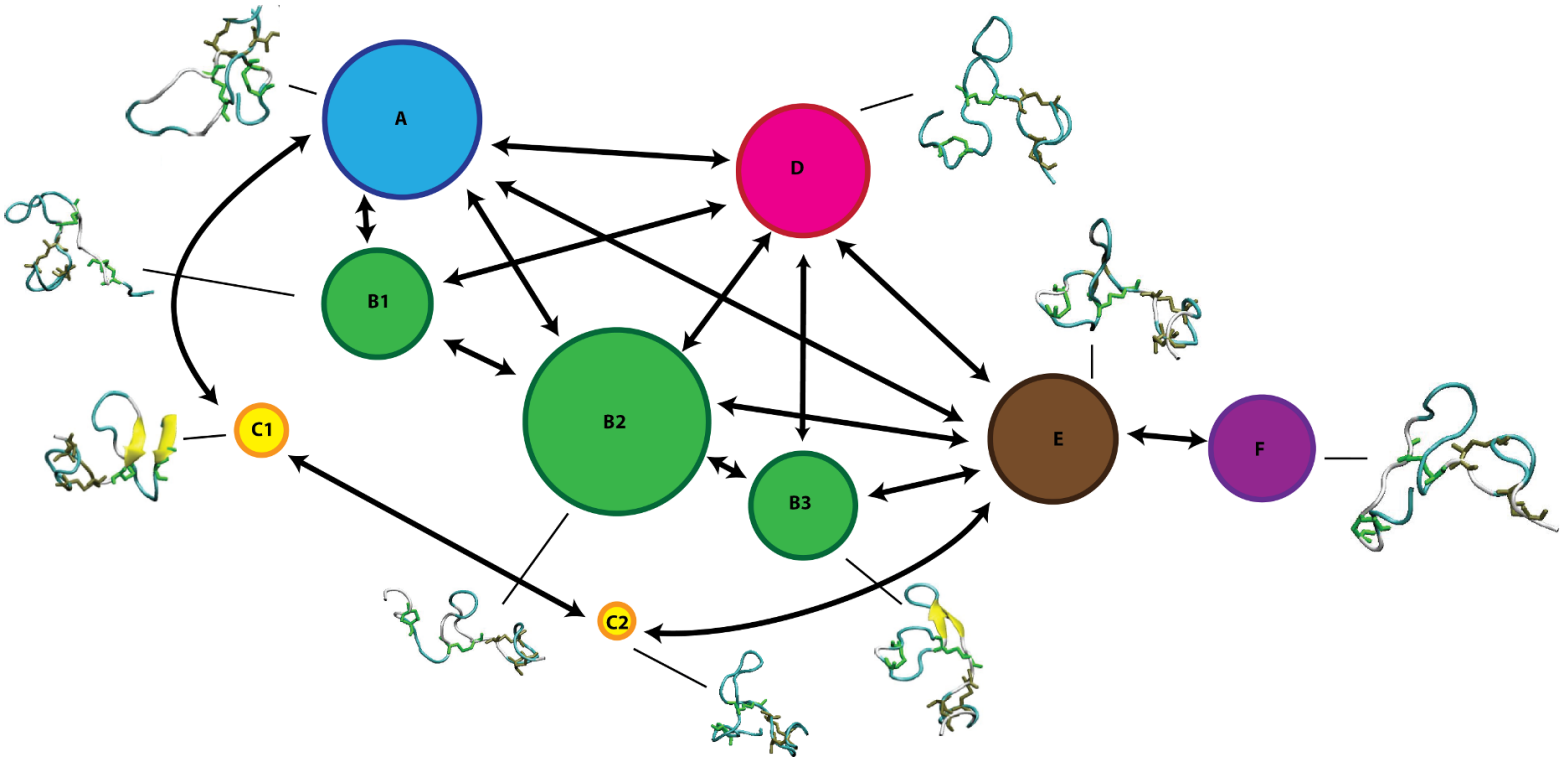
Markov State Model of Bliα peptide. MSM network is based on the information listed in Table 1. The 9 states are depicted as beads with size proportional to their occupation, arrows represent allowed transitions between states according to the corresponding transition matrix in Fig 9.

**Fig 7:**
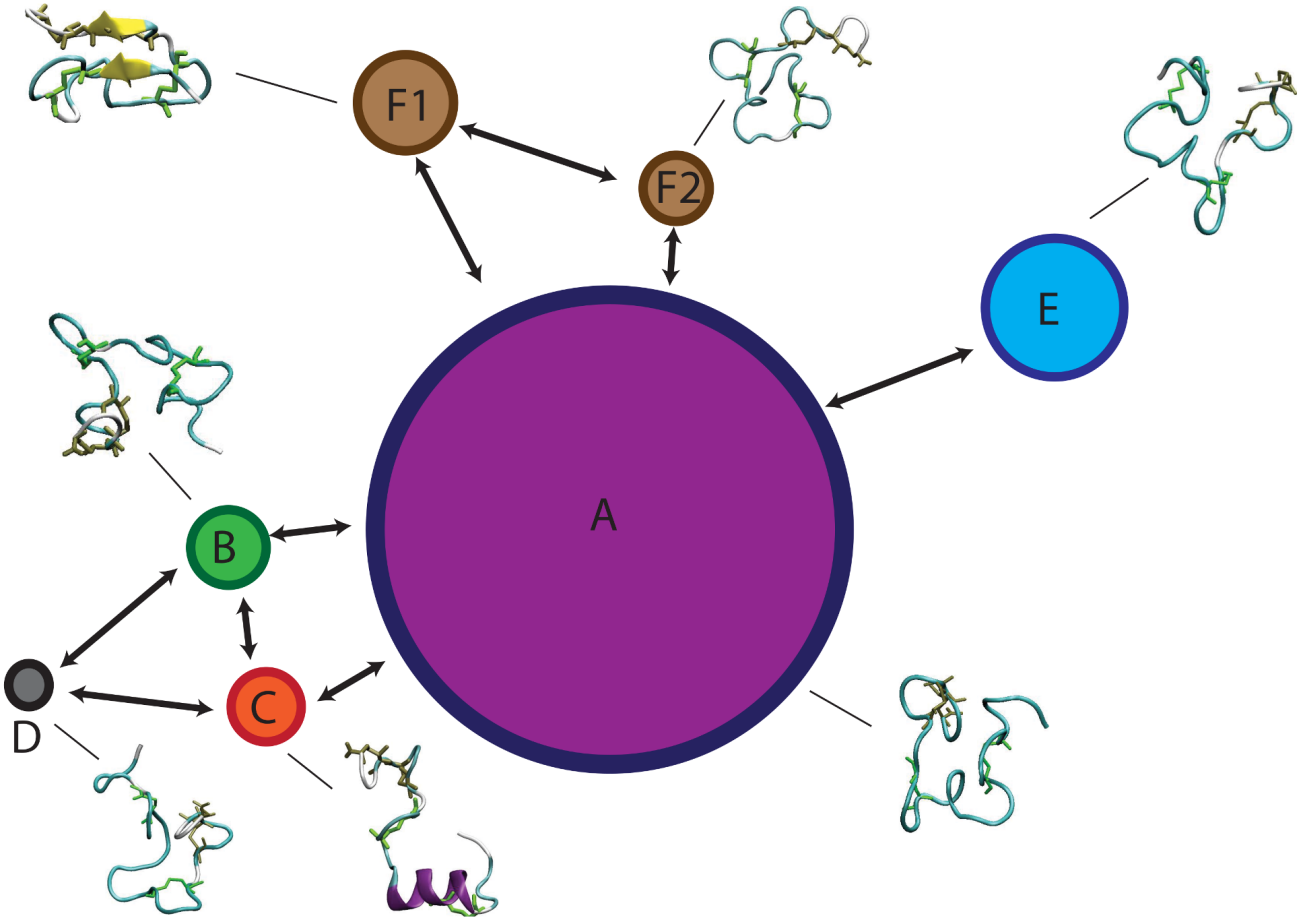
Markov State Model of Lchβ peptide. MSM network is based on the information listed in Table 1. The seven states are depicted as beads with size proportional to their occupation, arrows represent allowed transitions between states according to the corresponding transition matrix in Fig 10.

The MSMs of the three peptides were graphically represented as a network, where each state is depicted as a bead with a size proportional to its occupation, while the allowed transitions are shown as directed arrows. Each state is accompanied with the corresponding representative structure (Figs 5–7).

**Fig 8:**
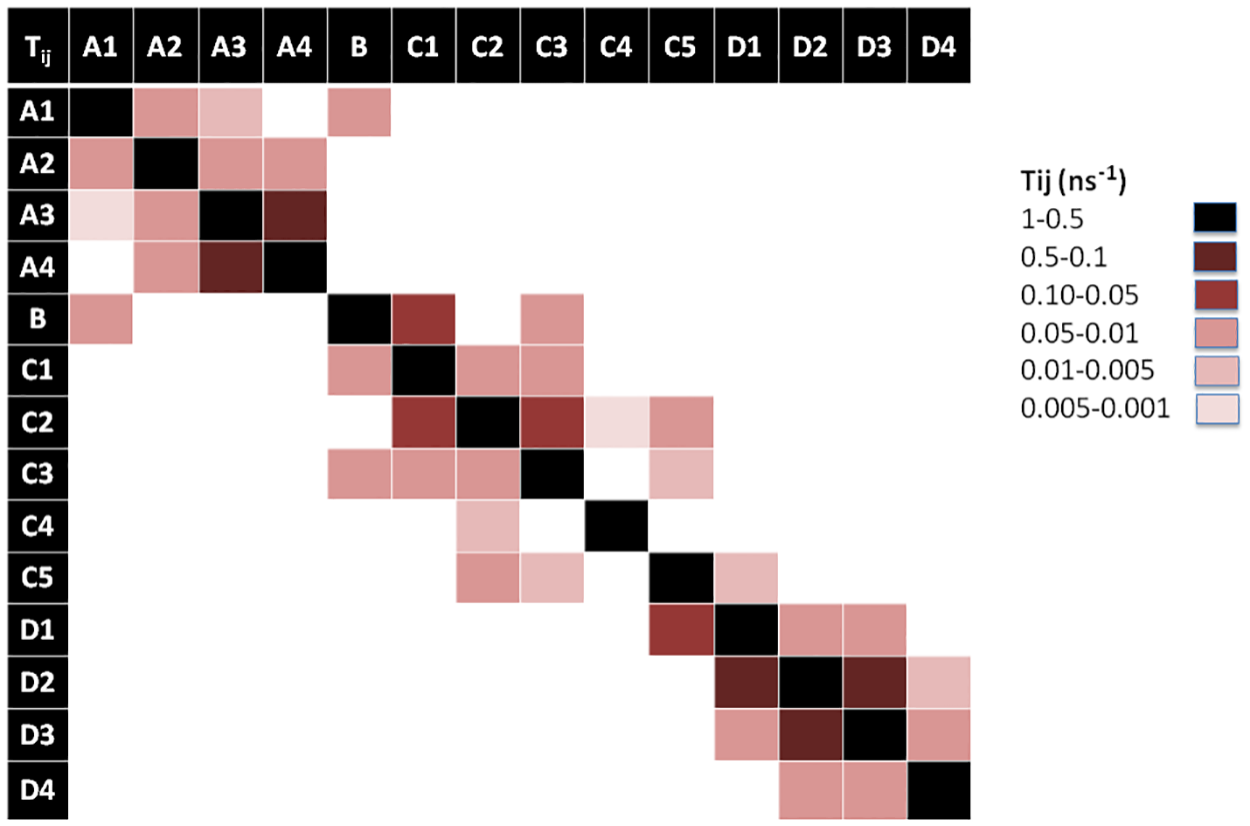
Transition matrix of Lchα peptide. The transition matrix elements are given in S2 File.

**Fig 9:**
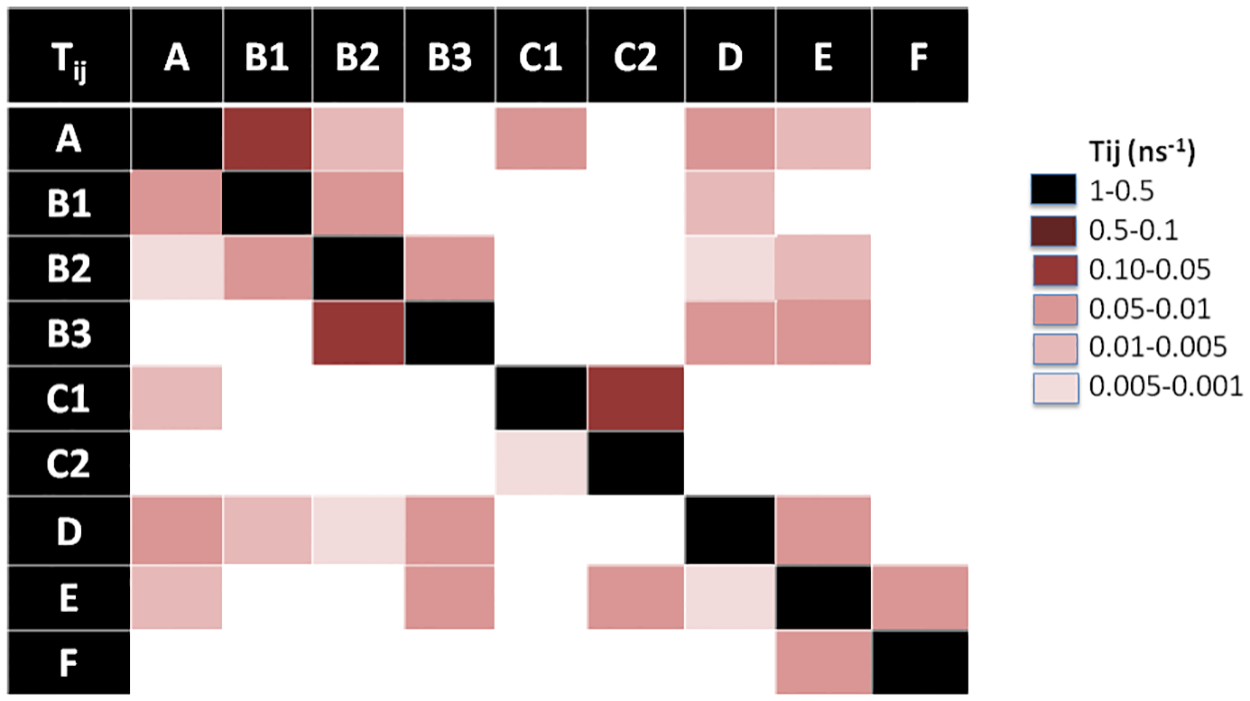
Transition matrix of Bliα peptide. The transition matrix elements are given in S2 File.

**Fig 10:**
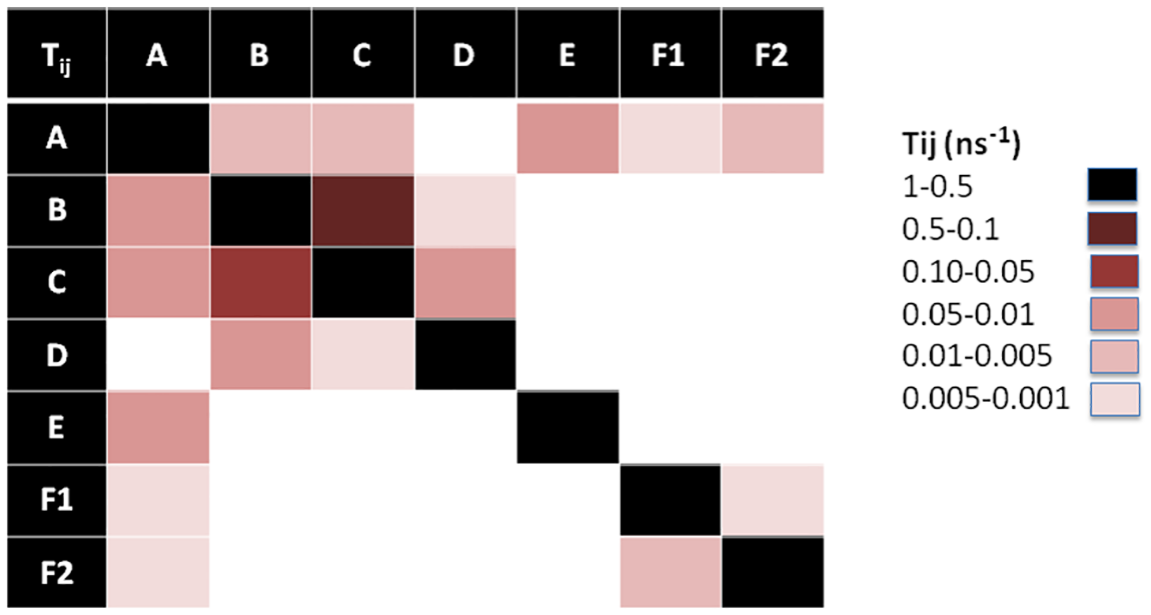
Transition matrix of Lchβ peptide. The transition matrix elements are given in S2 File.

Characterization of the MSM of each peptide is done in terms of 1) its population (*π*_*i*_) or weight of each state which can be intuitively associated with the “depth” of the associated free energy well (in the classical surface representation of free energy landscapes) or how likely is the state to be visited, 2) the average RMSD of all C_α_ with respect to the initial conformation, 3) the average escape time (*t*_*e*_), 4) the free energy differences (Δ*G*_*i*_) between state i and the most occupied state, 5) the corresponding entropy (S_i_) and the 6) the strength of average dipole moment (*μ*). These values are listed in Table 1. In addition, in order to gain a precise picture of the connectivity network and transition probabilities, graphic representations of the transition matrices T_ij_ are given for each peptide in Figs 8–10, respectively.

**Table 1:** MSM properties of the Lchα, Bliα and Lchβ peptides. Occupation of each state π_i_; root mean square deviation, RMSD; average escape time, t_e;_ free energy differences between states, ΔG_ij_ calculated with respect to the most occupied state; entropy, S; average of the dipolar moment, <μ>. Standard errors associated with each quantity are indicated in gray.

Macrostate	*π*_*i*_	RMSD (Å)	*t*_*e*_ (ns)	Δ*G*_*i*,_*/k*_*B*_*T*	S/k_B_	<*μ*> (D)
**Lchα**						
A1	0.047 ± 0.005	5.3± 1.3	30.0 ± 4.0	1.51 ± 0.02	0.194 ± 0.010	86.3 ± 22
A2	0.017 ± 0.003	2.3 ± 0.5	11.0 ± 0.4	2.55 ± 0.14	0.068 ± 0.009	104.0 ± 17
A3	0.009 ± 0.002	3.4 ± 1.7	2.3 ± 0.01	3.16 ± 0.01	0.042 ± 0.07	57.8 ± 24
A4	0.024 ± 0.003	5.2 ± 0.9	6.0 ± 0.6	2.18 ± 0.01	0.095 ± 0.008	82.1 ± 18
B	0.053 ± 0.005	5.1 ± 1.2	11.8 ± 0.7	1.39 ± 0.02	0.188 ± 0.021	92.7 ± 18
C1	0.054 ± 0.005	5.8 ± 1.0	4.5 ± 0.1	1.38 ± 0.02	0.157 ± 0.01	63.0 ± 27
**C2**	0.212 ± 0.010	7.3 ± 0.9	25.1 ± 6.0	0.00	0.552 ± 0.006	74.2 ± 21
C3	0.136 ± 0.08	7.1 ± 0.9	27.8 ± 0.2	0.64 ± 0.04	0.362 ± 0.008	67.3 ± 21
C4	0.072 ± 0.006	2.8 ± 0.9	143.0 ± 50.0	1.09 ± 0.03	0.189 ± 0.010	95.4 ± 15
C5	0.088 ± 0.007	4.9 ± 0.5	59.0 ± 12.0	0.88 ± 0.03	0.214 ± 0.010	75.1 ± 16
D1	0.051 ± 0.005	6.4 ± 1.0	5.0 ± 0.1	1.42 ± 0.02	0.240 ± 0.01	53.4 ± 23
D2	0.099 ± 0.007	6.5 ± 0.8	4.0 ± 0.1	0.77 ± 0.03	0.297 ± 0.009	49.8 ± 17
D3	0.056 ± 0.005	5.9 ± 0.8	3.0 ± 0.1	1.34 ± 0.02	0.189 ± 0.009	55.7 ± 19
D4	0.028 ± 0.004	4.3 ± 0.8	55.0 ± 12.0	2.04 ± 0.02	0.094 ± 0.01	70.8 ± 15
**Bliα**						
A	0.147 ± 0.009	8.0 ± 1.2	4.7 ± 0.2	0.11 ± 0.05	0.501 ± 0.008	66.3 ± 21
B1	0.130 ± 0.008	6.1 ± 1.1	8.0 ± 0.5	1.45 ± 0.05	0.389 ± 0.008	67.9 ± 22
**B2**	0.164 ± 0.009	4.3 ± 0.9	7.2 ± 0.4	0.00	0.414 ± 0.007	81.0 ± 20
B3	0.097 ± 0.007	6.7 ± 0.8	5.4 ± 0.8	0.53 ± 0.04	0.292 ± 0.009	82.1 ± 17
C1	0.052 ± 0.005	6.5 ± 0.5	16.7 ± 1.4	1.15 ± 0.03	0.186 ± 0.010	58.6 ± 18
C2	0.029 ± 0.004	4.2 ± 1.0	8.1 ± 0.3	1.73 ± 0.02	0.121 ± 0.010	59.0 ± 19
D	0.119 ± 0.008	5.6 ± 0.8	6.6 ± 0.3	0.32 ± 0.04	0.185 ± 0.009	65.3 ± 22
E	0.147 ± 0.009	5.0 ± 1.2	11.1 ± 1.0	0.11 ± 0.05	0.381 ± 0.008	75.0 ± 17
F	0.148 ± 0.009	6.2 ± 0.9	40.0 ± 12.0	0.10 ± 0.05	0.517 ± 0.008	90.3 ± 16
**Lchβ**						
**A**	0.534 ± 0.006	7.2 ± 0.9	7.8 ± 0.8	0.00	1.142 ± 0.006	74.3 ± 24
B	0.078 ± 0.009	6.9 ± 0.8	4.9 ± 0.1	1.92 ± 0.03	0.238 ± 0.009	70.3 ± 20
C	0.074 ± 0.005	7.2 ± 1.4	4.5 ± 0.1	1.98 ± 0.01	0.258 ± 0.014	69.7 ± 16
D	0.041 ± 0.009	5.5 ± 1.6	41.0 ± 7.0	2.57 ± 0.09	0.160 ± 0.011	92.2 ± 18
E	0.149 ± 0.009	8.8 ± 1.1	37.0 ± 10.0	1.28 ± 0.02	0.507 ± 0.008	57.9 ± 19
F1	0.087 ± 0.007	8.8 ± 0.4	173.1 ± 89.0	1.81 ± 0.01	0.254 ± 0.01	122.8 ± 13
F2	0.039 ± 0.004	1.4 ± 0.4	78.3 ± 23.0	2.62 ± 0.01	0.126 ± 0.009	123.5 ± 15

#### MSM of Lchα component

**Fig 11:**
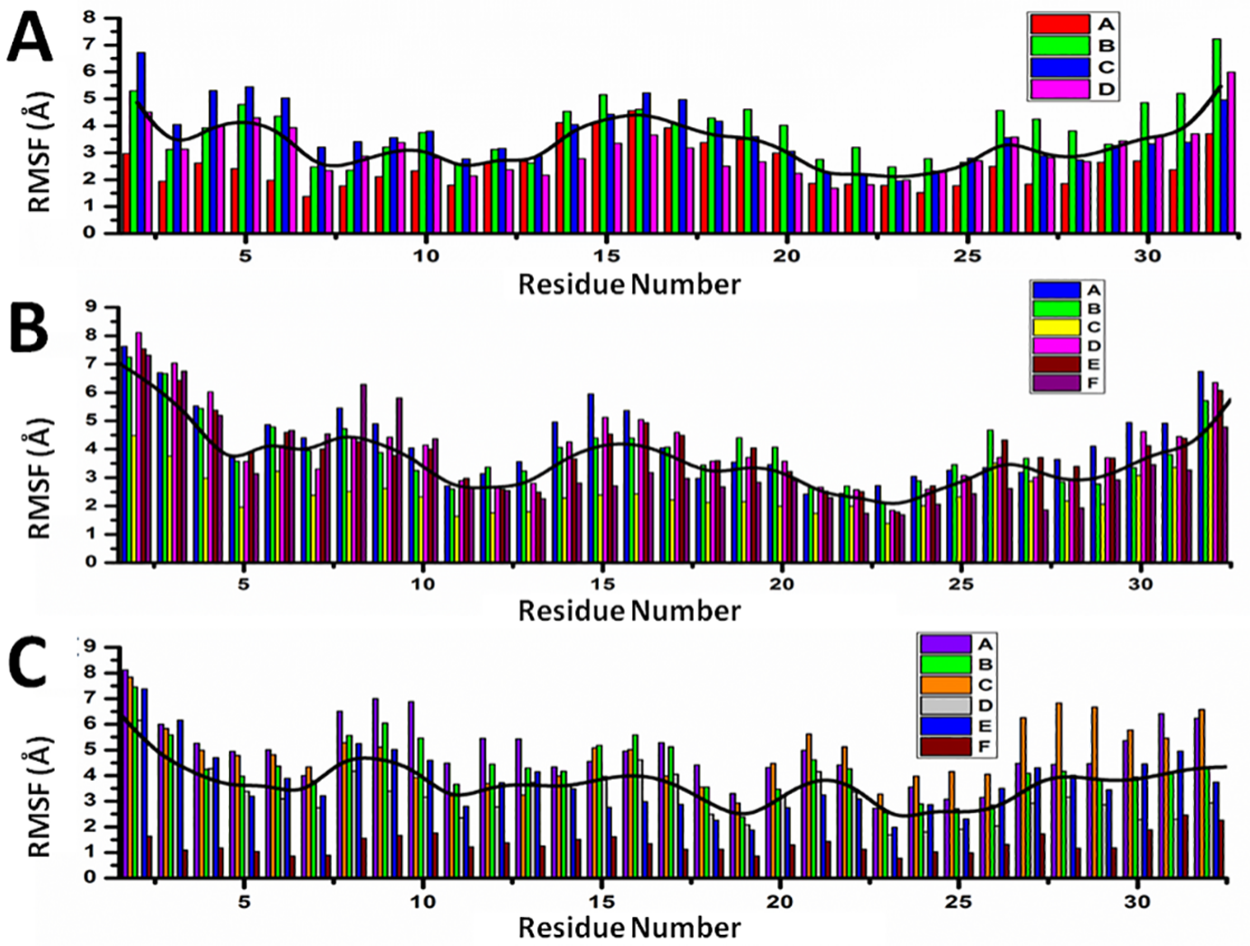
C_α_-Root mean square fluctuations for all residues of MSM states. (A) Lchα, (B) Bliα and (C) Lchβ. Black traces represent for each peptide the sum of the RMSF over all MSM states.

Fig 5 shows a graphic representation of the MSM for Lchα. The network shows three well differentiated regions with distinct kinetic properties labeled as A, C and D, with B playing the role of a transition state between regions A and C. Clustering of the MSM in four communities was performed with a modularity algorithm [46]. States in A are related to a first relaxation stage, where the peptide suffers different fast conformational changes before entering in the C region. These set of four states are characterized by low occupations and low RMSD values with respect to the initial structure, see Table 1 and Fig 5. The A-states represent 10% of the network population. While the state A3 shows a 3_10_ -helix structure involving ring C (residues 22 to 27), states A1 and A2 are characterized by an open and closed random-coil conformations. In contrast, state A4 exhibits an antiparallel β-sheet involving the residues 4–7 and 10–14. All these four communities are interconnected with each other through low energy barriers, as reflected by the relatively high values of the transition matrix elements depicted in Fig 8. In general the largest fluctuations within this subgroup of states are predicted for the fragment between residues 12 and 20, exactly at the central loop defining ring B, with RMSF values of up to 5 Å. While rings A, C and D are stabilized by the methyllanthionine bridges as reflected by the significantly lower RMSF values of ca. 2 Å. (Fig 11)

States in C can be grouped in five macrostates. These states constitute the main region of the equilibrium ensemble of the peptide encompassing 60% of the population. The peptide adopts a mainly coil structure as usually found for most AMP in water solution [13]. Only state C3 shows an antiparrallel β-sheet conformation including residues 12–13 and 19–20 at the central B-loop. Furthermore, states in C are strongly connected with relatively low free energy barriers between them as indicates by the non-negligible transition matrix elements in Fig 8. In this network, C2 is predicted with lowest the free energy and highest entropy, represents the main basin connecting all other states together. In contrast to states in A, states in C are characterized by a highly mobile N-terminal region reflected in RMSF-values between 4–7 Å for residues 1 to 7 (Fig 11).

State B, plays a crucial role in the peptides kinetic. This state acts as a bridge connecting communities A and C, as reflected by the non-negligible transition probability value (A1, B), (C1, B) and (C3, B) in Fig 8. In fact, removal of B from the network leaves A and C disconnected from each other. In contrast to states A1, C1 (random coils) and C3 (β-sheet), state B is characterized by an α-helical structure comprising residues 6 to 11 and represents only 5% of the network. Moreover, state B is predicted to be very flexible, in particular in the C-terminal region for which RMSF-values up to 7 Å are computed.

Finally, states in D constitute the second largest equilibrium ensemble involving 25% of the network population. Within this group, four macrostates can be identified on the basis of their kinetic properties. In all four D-states Lchα adopts a random coil conformations. Interestingly, despite the lack of secondary structural elements, the peptide in D seem to be more rigid than in states A, B and C, in particular in the central loop (ring B) region where the RMSF-values hardly reach 4 Å. The four D-states are strongly interconnected with each other through very fast transition as reflected by the low escape time (Table 1), being D1 the only bridge to the C-states.

#### MSM of Bliα component

A graphic representation of the MSM of Bliα is given in Fig 6. As mentioned above, the MSM of Bliα can be grouped in six communities. Compared with Lchα, the structure of the equilibrium ensemble of this peptide is quite different. The free energy landscape is constituted by a number of well-populated states, which are relatively short lived and highly connected between each other (Figs 6 and 9).

State A appears in the first stage of the simulation, however, it is visited several times afterwards as indicated by the non-zero transition probabilities to states B1, D, C1 and E (Fig 9). This initial state, characterized by a coil structure, represents only 15% of the network population. The escape time predicted for this state is very low indicating fast transitions and low free energy barrier to the neighbouring states. Furthermore, state A seems to be very mobile since RMSF-values between 6–7 Å are predicted at the N-terminal-, C-terminal and at the central B-ring region.

The B state community can be understood as a combination of three macrostates, B1, B2 and B3. These three states alone contribute to 37% of the network population. While in the macrostates B1 and B2, the peptide adopts a random coil conformation, a short β-sheet is formed between residues 11–13 and 18–20 (ring B) in the macrostate B3 as also found in state C3 of Lchα. Interestingly, these three states are characterized by an elongated N-terminus segment resulting from replacing the methyllanthionine bridge connecting residues 3 and 7 (found in Lchα) with a much shorter lanthionine between positions 5 and 7. Within the B-states, B2 is predicted with lowest free energy is thus the central state in the kinetics. In analogy to the C2 state of Lchα, the B2 state acts as a basin from which transitions to the A, B1, B3, D and E states are allowed, as indicated by the non-negligible transition probabilities. Furthermore, for the B-states we predict relatively low escape time (below 8 ns) reflecting their unsteadiness.

The peripheral group C is composed by the C1 and C2 macrostates. Both of them play an interesting role in the kinetics connecting macrostates placed far away from each other in the continuous space state. While the C1 state adopts a parallel β-sheet conformation involving the residues close to the N-terminal and half of the central loop in ring B (G18 -L20), the C2 state exhibits a closed coiled conformation. The escape-time associated to C1 (16.7 ns) is slightly higher in comparison to C2 (8.1 ns) reflecting higher energy barriers for the C1 state compared to C2. Analysis of the RMSF plots for Bliα in Fig 11, shows that the C-states are the most rigid states of Bliα with an average RMSF-value of ca. 3 Å. As expected, the most flexible regions are the two terminal fragments. From an energetic point of view, the C-states together with B1 state are computed with highest free energies which are at least 1 K_B_T higher than that of the reference state B2.

State D is structurally similar to macrostate A, characterized by relatively closed random coil conformation. In fact the RMSF-values along the entire peptide of these two states are very similar to each other, with peaks at the N- and C- terminal region and at residues forming ring B. State D is also well connected to the other macrostates by relatively low energy barriers as indicated by the non-negligible transition matrix elements (Fig 9) and the short escape time of only 6.6 ns.

States E and F are similar to state D. In these two states Bliα adopts a closed coil structure as in state D and each of them contribute to 15% of the population network. In other words, these three states are well populated. The RMSF plots in Fig 11, show that in both states the peptide is highly mobile although in state F the peptide’s flexibility between residues 7 and 10 is particularly large compared to all other states. The escape time predicted for these two states is significant, in particular that computed for state F of 40 ns (Table 1). These values reflect the high thermal stability of these states, which are also predicted with relatively low free energies compared to reference C2 state. Finally, the transition probability matrix of Bliα (Fig 9) indicates that, despite its low energy and high thermal stability, state F is poorly connected to the main network. Only one weak transition to state E is predicted.

#### MSM of Lchβ component

Fig 7 shows a graphic representation of the MSM of Lchβ. The MSM network consists of seven states grouped in six communities. Comparison with the MSM of the α-components shows distinct differences. In Lchβ the network is mainly characterized by a central macrostate A, with other peripheral states connected to it.

In state A, the peptide adopts a coil structure with the highest population of the system (53%). Here, the escape time is low enough (< 8 ns) to allow several transitions to neighboring states generating a well interconnected network. The escape time together with the large population, reflect low free energy barriers separating A from the other states. This probably means that state A can be considered as a wide shallow basin with low free energy and high entropy. Since state A is also predicted to be the lowest in free energy (2/K_B_T lower than other states), it is being considered as reference state for computing free energy differences. In this state the peptide is very flexible, especially in the ring A region (residues 7 to 10), with RMSF-values up to 7 Å. In analogy to the other two peptides, the N- and C- terminal regions are highly mobile.

Noteworthy are the macrostates C and F1. These are the only states where the peptide adopts well defined secondary structures. In the case of state C, an α-helix is built between residues 7 and 16, while in the case of the F1 state, a parallel β-sheet structure is formed between residue 5–6 and residues 26–27. Although these two states are practically isoenergetic, they are connected to each other only via the central basin (state A). Interestingly, their kinetic properties are completely different. While F1 is a strongly confined state with a mean escape time of 173 ns, the α-helical state C is surrounded by low energy barriers as reflected by the very low mean escape time of 4.5 ns allowing fast transitions to the neighboring states A, B and D. Furthermore, the F1 state is predicted as the most rigid macrostate of the entire network with RMSF below 2 Å (Fig 11) although the entropic contribution to the free energy does not differ significantly from the other cases.

The macrostates B, D and E are also described by various unordered structures. However, the three of them follow different kinetics and are characterized by distinct energetics. Among them state B seems to be the most interesting one. State B is very unstable with a mean escape time of 5 ns, low occupation (8%) and moderate transition rates. This state is practically isoenergetic with the α-helix state C (ΔG~1.9 K_B_T). The two of them are separated by a low energy barrier reflected by the high transition matrix elements T_BC_ and T_CB_ of 0.1 ns^-1^ and 0.2 ns^-1^. These two states clearly act as bridge connecting the main basin (state A) with the peripheral high energy state D. This state, D, is only seldomly visited (4% occupation), but once done, the peptide spends significantly more time inside it (mean time escapes of ca. 40 ns).

### Dynamics of Lchα, Bliα and Lchβ

The MSMs produced for the two α-compotents, Lchα and Bliα, differing in the primary structure of the N-terminal domain, are clearly dissimilar. At the structural level we could show that both peptides spend most of the time in various random coil conformations in aqueous solution in perfect agreement with experimental observations [16, 19, 20]. However, although both of them can fold in β-sheet structures, only Lchα is capable of adopting an α-helical conformation in the vicinity of ring A. The formation of the α-helix structure in Lchα seems to be stabilized by the presence of the methyllantionine bridge involving Ala7 and Abu3, which is absent in Bliα. In other words, only Lchα is capable of undergoing secondary structure transitions from α-helix to β-sheet, and vice versa, in aqueous solution. A very similar phenomenon has been predicted for Lchβ. In this case, the α-helical structure observed from NMR-spectroscopy converts into β-sheet involving N-terminal residues Dhb5-Dhb6 and the C-terminal Lys27-Ala28. Transitions between these two conformational states occur via transient random coil conformations. Interestingly, despite the tendency of the CHARMM force field to over-stabilize α-helical elements and the NMR studies which favor an α-helix conformation of the Lchββ peptide in methanol [16], the residence time in the ββ-sheet conformation (state F1) is one order of magnitude longer than in the αα-helical state (state C). Thus, the structural properties and dynamic behavior of the peptides in aqueous solution clearly differ from that observed via NMR spectroscopy in methanol solution.

The delicate equilibrium between random coil conformations, α-helical structure and β-sheet fold can be perturbed by the presence of a membrane, lipid molecules or any other reaction partner. Such secondary structure transitions in aqueous solution have been predicted and reported for other AMPs in the past [13, 47, 48], we extrapolate such observable also for the lantibiotics. While the folding of random coiled structure into α-helices or β-sheets upon interaction with lipids or membrane has not only been detected for many antimicrobial systems but it has been considered as an important process determining the mode of action [13, 47, 48].

Finally, the molecular dynamics simulation presented in this paper were performed with the CHARMM27 force field. This force field is widely used to accurately describe a large variety of protein systems in their folded state [49]. For unfolded short peptides, as the ones investigated in this work, benchmark studies using various protein force fields demonstrated that CHARMM27 in general overstabilizes the formation of helical structures [49]. Thus the secondary structure content of the MSMs generated within this work may be biased by the intrinsic deficiencies of the CHARMM27 force field. Given the lack of appropriate experimental structure information for the Lichenidicin peptides, the effect of the molecular mechanics force field on the structure and dynamic properties of the systems can only be evaluated by repeating the simulations using other protein force fields.

## Conclusions

Two variants of the two-component Lichenicidin lanthibiotics, produced by the VK21 and I89 strains of *B*. *Licheniformis*, were investigated by means on molecular dynamics simulations with an extended CHARMM27 force field combined with Markov State Models. The combination of these two approaches allowed the prediction and description of dynamic properties by means of rigorous statistical analysis of the molecular dynamics trajectories, thus enabling the evaluation of dynamic and kinetic features which are not reachable via conventional experimental techniques. The three peptides addressed in this work, namely Lchα and Lchβ from *B Licheniformis* VK21 and Bliα from *B*. *Licheniformis* I89, show high flexibility over the entire sequence, in particular at the N- and C-terminal fragments. The same behavior has been reported for Lchα in methanol solution [16]. The three peptides adopt random coil conformations in aqueous solution although formation of β-sheets as well as α-helical structures are possible. However, α-helical structures are only predicted for Lchα and Lchβ. In the case of Lchα, the formation of a helical structure may result from deficiencies of the CHARMM27 force field which overstabilizes α-helix folds. In any case, significant secondary structure changes of the three Lichenicidin peptides are predicted by our 2*μ*s-long simulations. Such structural versatility is a key property of AMPs and lantibiotics for an efficient adaption in various biological environments.

## Supporting information

S1 Fig NMR structures of Lchα and Lchβ. Spatial structures of Lchα and Lchβ derived from NMR spectroscopy in methanol solution (16) depicted as cartoons using the VMD visualisation software (31). Black representations correspond to the first conformer out twenty (depicted in gray) structures deposited in the pdb files 2KTN and 2KTO for Lchα and Lchβ, respectively. Residues involved in the lanthionine, methyllanthionine and thioester bridges are depicted as red lines.
(TIF)

S2 Fig Force field parameters. Dihedral scan of ψ dihedral N14-C7-C4-N3 and φ dihedral C7-C4-N3-C1of Dhb computed at MP2/6-31G* level (blue line) and at MM level using force field parameters used for MD simulations (green line). For comparison, the potential energy curves for ψ and φ predicted using exclusively ParamChem parameters (pink line) and the force field parameters suggested by Turpin et al. (red line) are showed.
(TIF)

S3 Fig Force field parameters. Dihedral scan of ψ dihedral N14-C7-C4-N3 and φ dihedral C7-C4-N3-C1of Dha computed at MP2/6-31G* level (blue line) and at MM level using force field parameters used for MD simulations (green line)
(TIF)

S4 Fig Convergence of dynamic models. Evolution of the total energy of three peptides: Lchα, Bliα and Lchβ in the course of 2 μs of simulation.
(TIF)

S5 Fig Time series of MSM macrostates for the 2μs MD simulation. Time series of macrostates predicted for Lchα, Bliα and Lchβ.
(TIF)

S6 Fig Validation: Chapman-Kolmogorov test. Implied timescales as a function of the lag time. Red: 1 ns, blue: 2 ns, green: 3 ns, brown: 5 ns and purple: 10 ns estimated for Lchα (left), Bliα (center) and Lchβ (right). The employed timescales are calculated as: t_i_ = τ/log(λ_i_), where λ_i_ are the eigenvalues of the transition matrix. The Markov state models in the manuscript are estimated with a lag time of 1ns, which is the sampling time employed in the MD simulations. Clearly, the timescales level for the employed lag time, validating the markovianity of the calculated models.
(TIF)

S7 Fig TICA spectrum for the three peptides. Plot of the TICA eigenvalues for growing magnitudes for Lchα (left), Bliα(center) and Lchβ(right). We represent our system by the linear subspace defined by the first three components. As seen in the plot, that covariance shows a strong nonlinear behavior and these three first components accounts for more than 50% of the fluctuations of the system.
(TIF)

S1 File Force field parameters. CHARMM- compatible force field for Dehydroalanine (Dha) and Dehydrobutyrine (Dhb) residues and force field parameters for lanthionine and methyllanthionine bridges.
(PDF)

S2 File MSM transition matrices. Transition matrices of MSM computed for the Lchα, Bliα and Lchβ.
(PDF)
