# Supplementary figures and images for: Structure, dynamics and kinetics of two-component Lantibiotic Lichenicidin
Source: PLoS One. 2017 Jun 27;12(6):e0179962. doi: 10.1371/journal.pone.0179962 (PMC5487065; doi:10.1371/journal.pone.0179962)

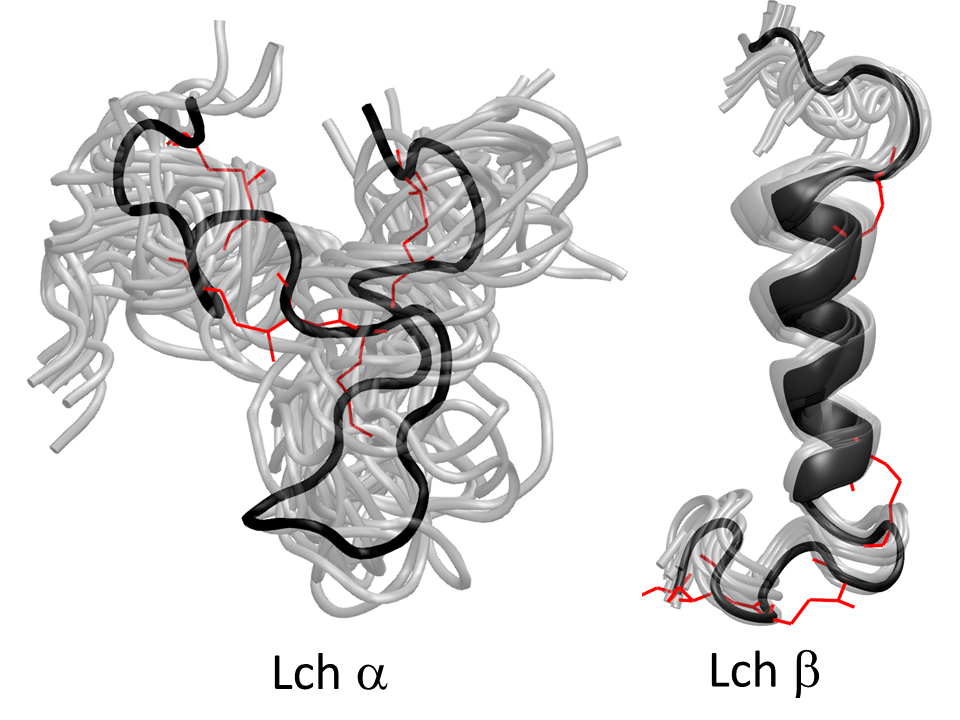

Supplement: S1 Fig — Spatial structures of Lchα and Lchβ derived from NMR spectroscopy in methanol solution (16) depicted as cartoons using the VMD visualisation software (31). Black representations correspond to the first conformer out twenty (depicted in gray) structures deposited in the pdb files 2KTN and 2KTO for Lchα and Lchβ, respectively. Residues involved in the lanthionine, methyllanthionine and thioester bridges are depicted as red lines. (TIF) [file pone.0179962.s001.tif]

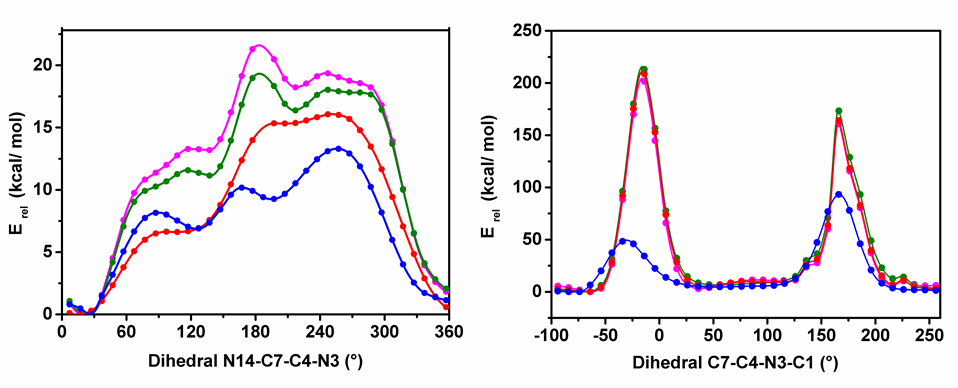

Supplement: S2 Fig — Dihedral scan of ψ dihedral N14-C7-C4-N3 and φ dihedral C7-C4-N3-C1of Dhb computed at MP2/6-31G* level (blue line) and at MM level using force field parameters used for MD simulations (green line). For comparison, the potential energy curves for ψ and φ predicted using exclusively ParamChem parameters (pink line) and the force field parameters suggested by Turpin et al. (red line) are showed. (TIF) [file pone.0179962.s002.tif]

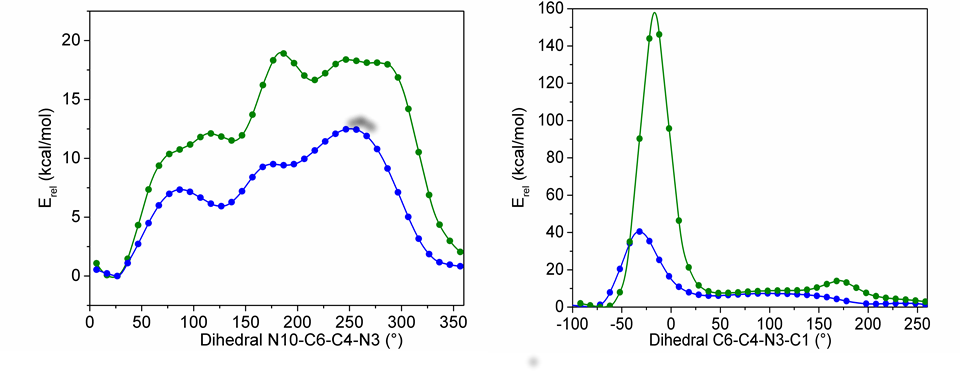

Supplement: S3 Fig — Dihedral scan of ψ dihedral N14-C7-C4-N3 and φ dihedral C7-C4-N3-C1of Dha computed at MP2/6-31G* level (blue line) and at MM level using force field parameters used for MD simulations (green line) (TIF) [file pone.0179962.s003.tif]

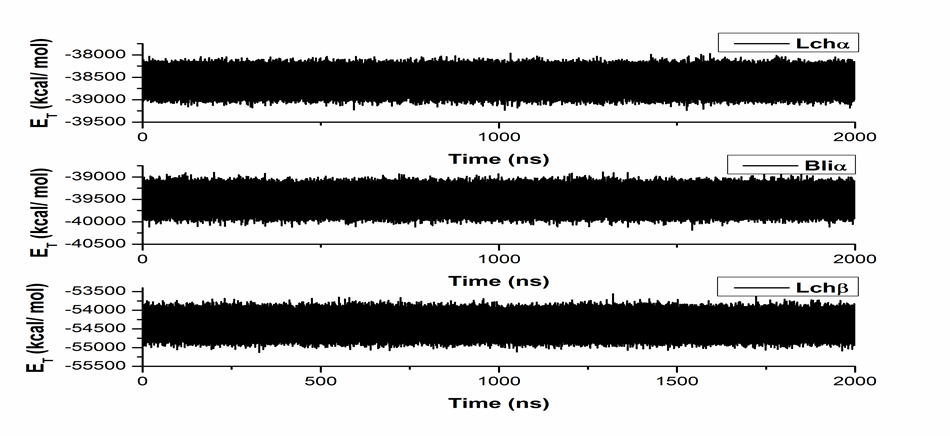

Supplement: S4 Fig — Evolution of the total energy of three peptides: Lchα, Bliα and Lchβ in the course of 2 μs of simulation. (TIF) [file pone.0179962.s004.tif]

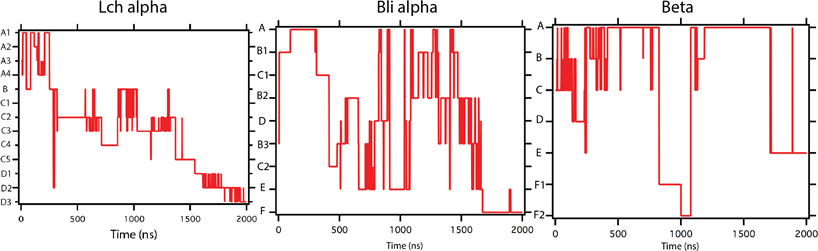

Supplement: S5 Fig — Time series of macrostates predicted for Lchα, Bliα and Lchβ. (TIF) [file pone.0179962.s005.tif]

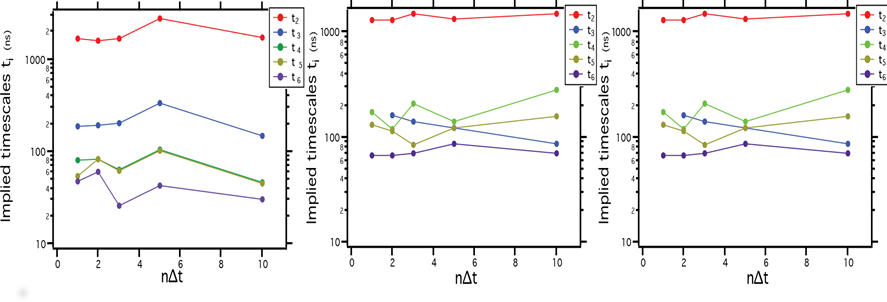

Supplement: S6 Fig — Implied timescales as a function of the lag time. Red: 1 ns, blue: 2 ns, green: 3 ns, brown: 5 ns and purple: 10 ns estimated for Lchα (left), Bliα (center) and Lchβ (right). The employed timescales are calculated as: ti = τ/log(λi), where λi are the eigenvalues of the transition matrix. The Markov state models in the manuscript are estimated with a lag time of 1ns, which is the sampling time employed in the MD simulations. Clearly, the timescales level for the employed lag time, validating the markovianity of the calculated models. (TIF) [file pone.0179962.s006.tif]

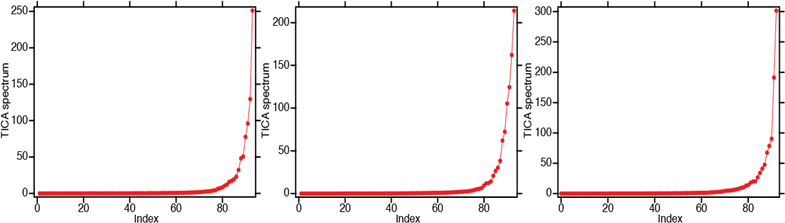

Supplement: S7 Fig — Plot of the TICA eigenvalues for growing magnitudes for Lchα (left), Bliα(center) and Lchβ(right). We represent our system by the linear subspace defined by the first three components. As seen in the plot, that covariance shows a strong nonlinear behavior and these three first components accounts for more than 50% of the fluctuations of the system. (TIF) [file pone.0179962.s007.tif]
