# Supplementary material for: Structure, dynamics and kinetics of two-component Lantibiotic Lichenicidin
Source: PLoS One. 2017 Jun 27;12(6):e0179962. doi: 10.1371/journal.pone.0179962 (PMC5487065; doi:10.1371/journal.pone.0179962)
Supplement: S1 File — CHARMM- compatible force field for Dehydroalanine (Dha) and Dehydrobutyrine (Dhb) residues and force field parameters for lanthionine and methyllanthionine bridges. (PDF) [file pone.0179962.s008.pdf]

S1 File. Force field parameters

1.1 Dha topology

```
RESI      DHA       0.00    !   2,3-Didehydroalanine
GROUP
ATOM      N         NH1                      -0,47  !
ATOM      HN        H                         0,31  !      |
ATOM      CA        CW                        0,16  !   HN-N     HB1
GROUP                                               !      |     /
ATOM      C         C                         0,51  !      CA==CB
ATOM      O         O                        -0,51  !      |     \
GROUP                                               !   O  = C     HB2
ATOM      CB        CEL2                     -0,42  !      |
ATOM      HB1       HEL2                      0,21  !
ATOM      HB2       HEL2                      0,21  !
BOND  N HN  N CA  CA C
BOND  C +N  CB  HB1  CB  HB2
DOUBLE  O C
DOUBLE  CA  CB
IMPR N -C CA HN C CA +N O
DONOR  HN N
ACCEPTOR  O C
AUTO ANGE  DIHE
```

1.2 Dhb topology

```
RESI      DBU       0.00     ! DHB (Z)-2,3-didehydrobutyrine
GROUP
ATOM      N         NH1       -0,47  !
ATOM      HN        H          0,31  !
ATOM      CA        CW         0,16  !
GROUP                                !
ATOM      C         C          0,51  !      |
ATOM      O         O         -0,51  !   HN-N     HB1
GROUP                                !      |     /
ATOM      CB        CEL1      -0,15  !      CA==CB
ATOM      HB        HEL1       0,15  !      |     \
GROUP                                !   O  = C     CG--HG1
ATOM      CG        CTL3      -0,27  !      |        /   \
ATOM      HG1       HAL3       0,09  !             HG3   HG2
ATOM      HG2       HAL3       0,09  !
ATOM      HG3       HAL3       0,09  !
BOND CG CB CA N HN N
BOND C CA C +N CB HB
BOND CG HG1 CG HG2 CG HG3
DOUBLE O C
```

```
DOUBLE CA CB
IMPR N -C CA HN C CA +N O
DONOR HN N
ACCEPTOR O C
AUTO ANGE DIHE
```

1.3 Remarks on atom-type assignment

Dehydroamino acids have a double bond between CA and CB, therefore in analogy to R-C=CH2 alkane groups in lipids, CB in Dha was assigned to a CEL2 atom type while HB1 and HB2 to HEL2. In the case of Dhb, CB and HB were assigned to a CEL1 and HEL1 atom types. The force field parameters associated to these atom types are available in the top_all36_lipid.rtf and the par_all36_lipid.prm files of the CHARMM36 force field. The amide- and carbonyl- groups of the backbones were assigned to the atom types NH1, H and C, O as done for all other canonical amino acids in the CHARMM force field. Finally, the CA atoms were assigned to the CG2D1O atom type from the CHARMM General Force Field v2b4 with a charge of 0.16, as suggested by Paramchem.org server for both molecules. This atom type was renamed to CW.

1.4 Force field parameters

The FF parameters used during the MD simulations for the dehydroamino acids were taken from the par_all36_protein.prm and par_all36_lipid.prm file of the CHARMM force field. Additional FF parameters associated to the atom type CW are listed below. Most of these values were generated with the Paramchem.org server or by analogy as indicated below. FF parameters for dihedral contributions modified according to Thormann & Hofmann study are written in italics.

```
BONDS
CW   C     250.000     1.4900  !  from CT1 C Ala Dipeptide
CW   CEL1  440.000     1.340   ! butene, from bond N C
NH1  CW    320.000     1.4300  ! from NH1 CT1
CW   CEL2  500.000     1.342   ! from CEL1 CEL2

ANGLES
CW   NH1  C      50.000   120.0000 ! from CG2D1O-NG2S1-CG201, penalty 21
H    NH1  CW     35.000   117.0000 ! from H-NH1-CT1, NMA Vibrational Modes (LK)
N    C    CW     80.000   116.5000 ! from CG2D1O CG201 NG2S0, penalty 4,5
NH1  CW   C      50.000   107.0000 ! from NH1-CT1-C Alanine Dipeptide
O    C    CW     80.000   121.0000 ! from O-C-CT1 Alanine Dipeptide
C    CW   CEL1   48.00    123.50   ! from CG2DC1-CG2D1O-CG201, penalty 40
CEL1 CW   NH1    60.00    122.00   ! from CG2DC1-CG2D1O-NG311, penalty 66
CEL2 CW   NH1    80.00    128.00   ! from propene CEL2 CEL1 CTL2
CEL2 CW   C      60.00    120.00   ! 1-butene; from CEL2 CEL1 CTL2
HEL1 CEL1 CW     52.00    119.50   ! 2-butene
HEL2 CEL2 CW     45.00    120.50   ! propene, from HEL2 CEL2 CEL1

DIHEDRALS
C    CW   NH1  C       0.4800  2   180.00 ! from X-CUA1-NP-X, Thormann & Hofmann 1998
CW   C    N    CP1     1.6000  1     0.00 ! from CG2D1O-CG201-NG2S0-CG3C51,penalty 24,4
CW   C    N    CP1     2.5000  2   180.00 ! from CG2D1O-CG201-NG2S0-CG3C51,penalty 24,4
CW   C    N    CP3     1.6000  1     0.00 ! from CG2D1O-CG201-NG2S0-CG3C52,penalty 24,4
CW   C    N    CP3     2.5000  2   180.00 ! from CG2D1O-CG201-NG2S0-CG3C52,penalty 24,4
CW   C    NH1  CT1     1.6000  1     0.00 ! from CT1-C-NH1-CT1
CW   C    NH1  CT1     2.5000  2   180.00 ! from CT1-C-NH1-CT1
CW   C    NH1  CW      1.6000  1     0.00 ! from CT1-C-NH1-CT1
CW   C    NH1  CW      2.5000  2   180.00 ! from CT1-C-NH1-CT1
CT1  C    NH1  CW      1.6000  1     0.00 ! from CT1-C-NH1-CT1
CT1  C    NH1  CW      2.5000  2   180.00 ! from CT1-C-NH1-CT1
CEL1 CW   NH1  C       1.8000  1     0.00 ! from CT2-CT1-NH1-C
CEL1 CW   NH1  C       0.4800  2   180.00 ! from CT2-CT1-NH1-C
H    NH1  C    CW      2.5000  2   180.00 ! from H-NH1-C-CT1
H    NH1  CW   C       0.4800  2   180.00 ! from H-NH1-CT1-C
HEL2 CEL2 CW   C       5.2000  2   180.00 ! from HE2-CE2-CE1-CT2
NH1  C    CW   NH1     0.6000  1     0.00 ! from NH1-C-CT1-NH1
NH1  CW   C    N       0.5000  2   180.00 ! from NG2S1-CG2D1O-CG201-NG2S0, penalty 111
NH1  CW   C    N       0.3500  3   180.00 ! from NG2S1-CG2D1O-CG201-NG2S0, penalty 111
NH1  CW   C    N       0.4000  6     0.00 ! from NG2S1-CG2D1O-CG201-NG2S0, penalty 111
```

```
O    C    CW   NH1      0.0000  1     0.00 ! from O-C-CT1-NH1
O    C    NH1  CW       2.5000  2   180.00 ! from O-C-NH1-CT1
O    C    CW  CEL1      0.7000  1   180.00 ! from OG2D1-CG201-CG2DC1-CG2DC1, penalty 40
O    C    CW  CEL1      1.2000  2   180.00 ! from OG2D1-CG201-CG2DC1-CG2DC1, penalty 40
O    C    CW  CEL1      0.1000  3   180.00 ! from OG2D1-CG201-CG2DC1-CG2DC1, penalty 40
O    C    CW  CEL1      0.2000  4     0.00 ! from OG2D1-CG201-CG2DC1-CG2DC1, penalty 40
C    CW   CEL1 HEL1     1.0000  2   180.00 ! from CTL3-CEL1-CEL1-HEL1
NH1  CW   CEL1 CTL3     2.5000  2   180.00 ! from NG311-CG2D1O-CG2DC1-CG321, penalty 67
H    NH1  CW   CEL1     0.4800  2   180.00 ! from X-CUA1-NP-X,Thormann & Hofmann 1998
CW   CEL1 CTL3 HAL3     0.0300  3     0.00 ! from CEL1-CEL1-CTL3-HAL3
CEL1 CW   C    N        0.700   1     0.00 ! from CG2DC1-CG2DC1-CG201-NG2S1, penalty 41
CEL1 CW   C    N        1.200   2   180.00 ! from CG2DC1-CG2DC1-CG201-NG2S1, penalty 41
CEL1 CW   C    N        0.100   3     0.00 ! from CG2DC1-CG2DC1-CG201-NG2S1, penalty 41
CEL1 CW   C    N        0.150   4     0.00 ! from CG2DC1-CG2DC1-CG201-NG2S1, penalty 41
CEL1 CW   C    NH1      0.700   1     0.00 ! from CG2DC1-CG2DC1-CG201-NG2S1, penalty 41
CEL1 CW   C    NH1      1.200   2   180.00 ! from CG2DC1-CG2DC1-CG201-NG2S1, penalty 41
CEL1 CW   C    NH1      0.100   3     0.00 ! from CG2DC1-CG2DC1-CG201-NG2S1, penalty 41
CEL1 CW   C    NH1      0.150   4     0.00 ! from CG2DC1-CG2DC1-CG201-NG2S1, penalty 41
CEL2 CW   C    NH1      1.800   2   180.00 ! from CG2R62-CG2R62-CG2R63-NG2R61
C    NH1  CW   CEL2     1.600   1     0.00 ! from CG2DC1-CG2O1-NG2S1-CG2R61
C    NH1  CW   CEL2     0.480   2   180.00 ! from CG2DC1-CG2O1-NG2S1-CG2R61, modified
                                           !  according to Thormann & Hofmann 1998
NH1  CW   CEL2 HEL2     5.000   2   180.00 ! from CG2DC2 CG2DC1 CG2DC3 HGA5
NH1  CW   CEL1 HEL1     3.00    2   180.00 ! from OG301-CG2D1O CG2DC1 HGA4
H    NH1  CW   CEL2     0.480   2   180.00 ! from X-CUA1-NP-X,Thormann & Hofmann 1998
O    C    CW   CEL2     0.700   1   180.00 ! from OG2D1-CG201-CG2DC1-CG2DC1,
O    C    CW   CEL2     1.200   2   180.00 ! from OG2D1-CG201-CG2DC1-CG2DC1
O    C    CW   CEL2     0.100   3   180.00 ! from OG2D1-CG201-CG2DC1-CG2DC1
O    C    CW   CEL2     0.200   4     0.00 ! from OG2D1-CG201-CG2DC1-CG2DC1
C    CW   CEL1 CTL3     0.56    1   180.00 ! from CG201-CG2DC1-CG2DC1-CG331
```

1.5 Topology files and additional force field parameters for the lanthionine and methyllanthionine bridges

```
PRES MLAN   0.00! path for methyllanthionine.
                ! follow with AUTOgenerate ANGLES DIHEDRALS command
GROUP
ATOM  1CB   CT2     -0.09!

ATOM  1HB2  HA       0.09!                   1HB2         2HB1
!ATOM 1HB3  HA     0.09!                     |            |
ATOM  1CG   CT3     -0.27!                   --1CB---2SG---2CB--
ATOM  1HG1  HA       0.09!                      |            |
ATOM  1HG2  HA       0.09!              1HG3--1CG--1HG1  2HB2
ATOM  1HG3  HA       0.09!                     |
GROUP                  !                    1HG2
ATOM  2CB   CT2     -0.11!
ATOM  2HB1  HA       0.09!
ATOM  2HB2  HA     0.09!
ATOM  2SG   S       -0.07!
!ATOM 2HG1  HS     0.16!
DELETE ATOM 1HB3
DELETE ATOM 2HG1
BOND 1CB 2SG
```

```
PRES LAN    0.00! path for Lanthionine.
                  ! follow with AUTOgenerate ANGLES DIHEDRALS command
GROUP
ATOM  1CB   CT2     -0.18!
ATOM  1HB1  HA       0.09!         1HB2    2HB1
ATOM  1HB2  HA       0.09!         |          |
!ATOM 1HB3  HA       0.09!         --1CB--2SG--2CB--
GROUP                    !         |          |
ATOM  2CB   CT2     -0.11!         1HB1        2HB2
ATOM  2HB1  HA       0.09!
ATOM  2HB2  HA       0.09!
```

```
ATOM  2SG   S       -0.07!
!ATOM 2HG1  HS    0.16!
DELETE ATOM 1HB3
DELETE ATOM 2HG1
BOND 1CB 2SG
```

DIHEDRALS

```
HA   CT2  S    CT2      0.2800  3     0.00
CT2  S    CT2  CT1      0.2400  1   180.00 ! from CG321 CG321 SG311 CG321
CT2  S    CT2  CT1      0.3700  3     0.00 ! from CG321 CG321 SG311 CG321
S    CT2  CT3  HA       0.1600  3     0.00 ! from SG311 CG321 CG331 HGA3
HB   CT1  CT2  S        0.1950  3     0.00 ! from HGA1 CG311 CG321 SG311
C    CT1  CT2  S        0.2000  3     0.00 ! from CG2O1 CG311 CG321 SG311
NH1  CT1  CT2  S        0.2000  3     0.00 ! from NG2S1 CG311 CG321 SG311
CT1  CT2  S    CT2      0.2400  1   180.00 ! from CG321 CG321 SG311 CG321
CT1  CT2  S    CT2      0.3700  3     0.00 ! from CG321 CG321 SG311 CG321
CT3  CT2  S    CT2      0.2400  1   180.00 ! from CG331 CG321 SG311 CG331
CT3  CT2  S    CT2      0.3700  3     0.00 ! from CG331 CG321 SG311 CG331
```
