# Supplementary material for: Structure, dynamics and kinetics of two-component Lantibiotic Lichenicidin
Source: PLoS One. 2017 Jun 27;12(6):e0179962. doi: 10.1371/journal.pone.0179962 (PMC5487065; doi:10.1371/journal.pone.0179962)
Supplement: S2 File — Transition matrices of MSM computed for the Lchα, Bliα and Lchβ. (PDF) [file pone.0179962.s009.pdf]

S2 File. MSM Transition Matrices

2.1 Transition Matrix for Lchα

T_{ij}	A1	A2	A3	A4	B	C1	C2	C3	C4	C5	D1	D2	D3	D4
A1	0.967	0.030	[illegible]		0.028									
A2	0.021	0.909	0.020	0.021										
A3	[illegible]	0.030	0.556	0.125										
A4		0.030	0.343	0.833										
B	0.025				0.915	0.059		0.025						
C1					0.024	0.778	0.013	0.021						
C2						0.089	0.950	0.070	[illegible]	0.014				
C3					0.014	0.020	0.046	0.954		0.010				
C4							0.009		0.993					
C5							0.018	0.008		0.983	0.006			
D1										0.097	0.8	0.030	0.031	
D2											0.150	0.750	0.272	0.007
D3											0.045	0.162	0.667	0.011
D4												0.018	0.012	0.982

Tij (ns^{-1})
- 1-0.5
- 0.5-0.1
- 0.10-0.05
- 0.05-0.01
- 0.01-0.005
- 0.005-0.001

2.2 Transition Matrix for Bliα

T_{ij}	A	B1	B2	B3	C1	C2	D	E1	F
A	0.837	0.071	0.008		0.047		0.043	0.008	
B1	0.090	0.875	0.012				0.007		
B2	0.009	0.035	0.861	0.091			[illegible]	0.007	
B3			0.095	0.865			0.019	0.040	
C1	0.009				0.940	0.071			
C2					0.004	0.877			
D	0.049	0.009	0.003	0.020			0.898	0.020	
E1	0.009			0.045		0.047	0.005	0.910	0.05
F								0.030	0.995

Tij (ns^{-1})
- 1-0.5
- 0.5-0.1
- 0.10-0.05
- 0.05-0.01
- 0.01-0.005
- 0.005-0.001

2.3 Transition Matrix for Lchβ.

T_{ij}	A	B1	B2	B3	C1	C2	D	E1	F
A	0.837	0.071	0.008		0.047		0.043	0.008	
B1	0.090	0.875	0.012				0.007		
B2	0.009	0.035	0.861	0.091			[illegible]	0.007	
B3			0.095	0.865			0.019	0.040	
C1	0.009				0.940	0.071			
C2					0.004	0.877			
D	0.049	0.009	0.003	0.020			0.898	0.020	
E1	0.009			0.045		0.047	0.005	0.910	0.05
F								0.030	0.995

Tij (ns^{-1})
- 1-0.5
- 0.5-0.1
- 0.10-0.05
- 0.05-0.01
- 0.01-0.005
- 0.005-0.001
